# Behavior Change Content and Implementation of Large Language Model–Driven Conversational Agents in Cardiometabolic Care: Scoping Review

**DOI:** 10.2196/89190

**Published:** 2026-07-15

**Authors:** Yuhan Zhao, Rongrong Guo, Yiqun Miao, Yuan Luo, Huiying Wang, Ying Wu

**Affiliations:** 1 School of Nursing, Capital Medical University, 10 You-An-Men Wai Xi Tou Tiao, Fengtai District, Beijing, 100069, China, 86 13910789837

**Keywords:** large language models, conversational agents, chatbots, cardiometabolic diseases, behavior change techniques, self-management, hybrid systems, implementation, transparency, scoping review

## Abstract

**Background:**

Large language models (LLMs) are increasingly embedded in conversational agents for cardiometabolic care. These systems could support self-management, but their behavior change content, delivery mechanisms, and implementation transparency are poorly understood.

**Objective:**

This scoping review mapped behavior change techniques (BCTs) used in LLM-driven conversational agents for cardiometabolic prevention and management, described how these techniques are delivered across static, rule-based, and generative mechanisms, examined LLM design, personalization, and safety reporting, and summarized user experience and behavioral or clinical outcomes.

**Methods:**

We searched PubMed, Web of Science, Embase, CINAHL, APA PsycInfo, IEEE Xplore, ACM Digital Library, arXiv, ClinicalTrials.gov, and the WHO International Clinical Trials Registry Platform for records published from January 1, 2020, to November 30, 2025. The final search was run on March 25, 2026, using this publication-date limit. Eligible studies reported a patient-facing text- or voice-based cardiometabolic conversational agent using an LLM or other transformer-based generative model. Two reviewers independently screened records and extracted data. BCTs were coded using the Behavior Change Technique Taxonomy v1; selected self-management BCTs were classified as static, rule-based or templated, or generative or context-aware. Empirical human-participant– or evaluator-based studies were appraised with the Mixed Methods Appraisal Tool, and a study-specific checklist assessed LLM implementation reporting transparency.

**Results:**

Thirty-eight studies were included; 19 involved empirical human-participant– or evaluator-based assessments, whereas 19 were technical and system-level evaluations, including framework-development, simulated-output, and proof-of-concept studies. Studies were concentrated in 2024‐2025. Instruction on how to perform behavior was identified in 30 of 38 (79%) studies, information about health consequences in 27 of 38 (71%) studies, and feedback and monitoring techniques in 19 of 38 (50%) studies. Most agents were positioned as educators or coaches targeting type 2 diabetes, obesity, or related cardiometabolic risk, and GPT-family models embedded in hybrid architectures with retrieval-augmented generation or rule-based components predominated. Generative outputs were used mainly for tailored explanations, risk information, and socioemotional responses, whereas self-monitoring, reminders, and structured interactions were more often rule-based or mixed-mode. Only 13 of 38 (34%) studies fully reported prompts or system messages, and 16 of 38 (42%) studies fully reported safety or oversight mechanisms. User evaluations reported good usability and perceived helpfulness, but behavioral or physiological outcomes were sparse and usually limited to pilot, short-term, or single-case designs.

**Conclusions:**

LLM-driven conversational agents for cardiometabolic care are proliferating but remain early-stage and methodologically heterogeneous. Current systems primarily use LLMs as educational and explanatory layers with “synthetic empathy” over rule-based data capture and safety functions, while behavior change content remains dominated by information provision and simple feedback. More rigorous comparative studies with longer follow-up are needed before firm conclusions can be drawn about sustained behavioral or clinical benefit.

## Introduction

### Rationale

Cardiometabolic conditions such as type 2 diabetes, hypertension, cardiovascular disease, heart failure, and obesity are among the leading causes of morbidity, mortality, and health care expenditure worldwide [1,2]. Effective management depends not only on pharmacotherapy but also on sustained changes in diet, physical activity, medication adherence, self-monitoring, and timely help seeking [3,4]. These behaviors are difficult to initiate and maintain in routine care, particularly in settings with constrained clinician time and limited access to specialized education or coaching services [5,6]. There is therefore substantial interest in scalable digital interventions that can provide ongoing, low-cost support for cardiometabolic self-management outside traditional clinical encounters [7,8].

Conversational agents and chatbots have emerged as a prominent class of digital tools for behavior change and self-management support [9,10]. Earlier generations relied largely on rule-based decision trees, scripted dialogues, and fixed message libraries [11]. More recently, large language models (LLMs) and generative AI have begun to reshape the technical foundations of conversational delivery [12,13]. Foundation models such as GPT-3.5 (OpenAI), GPT-4 (OpenAI), and other transformer-based systems can generate more flexible, context-sensitive responses, integrate free-text patient input, and be combined with retrieval-augmented generation (RAG) or other modules for data access and decision support [14-16]. Early demonstrations suggest that LLM-driven agents can answer cardiometabolic health questions, explain risk and treatment options, generate personalized lifestyle suggestions, and simulate supportive coaching dialogues [17-19]. At the same time, concerns have been raised about hallucinated content, inconsistent reasoning, bias, overconfidence, and opaque implementation choices, prompting calls for cautious, well-documented deployment of generative systems in health care [20,21].

From a behavioral medicine perspective, it is not sufficient to know that these systems are conversational or generative; it is also necessary to understand what behavior change content they actually deliver. The Behavior Change Technique Taxonomy v1 (BCTTv1) provides a structured way to specify the active ingredients of behavior change interventions across 93 hierarchically organized techniques [22]. Explicit identification of behavior change techniques (BCTs) can improve intervention transparency, facilitate replication and synthesis, and support more systematic optimization of digital health tools [23]. Applying this lens to LLM-driven cardiometabolic agents may help distinguish systems that primarily provide information from those that more deliberately support self-management, motivation, and longitudinal behavior change.

Despite the pace of development, the emerging evidence base for LLM-driven conversational agents in cardiometabolic care remains fragmented [9,24]. Studies span a continuum that includes technical and system-level evaluations, framework-development papers, simulated-output studies, expert- or user-based assessments, feasibility pilots, and early randomized evaluations [9,25]. They also vary widely in how clearly they describe the underlying models, prompts, safety guardrails, and personalization logic [11]. It also remains unclear which BCTs are embedded in these systems, how those techniques are operationalized through static messages, rule-based templates, or generative outputs, how transparently implementation details are reported, and what has been reported regarding user experience and preliminary behavioral or clinical outcomes.

### Objectives

Because this literature is heterogeneous in study design, intervention maturity, and reporting depth, and because the field is still at an early stage of development, a scoping review was undertaken to map the landscape rather than to estimate pooled effect sizes [7,26]. Accordingly, this review aimed to provide a structured overview of patient-facing LLM-driven conversational agents designed for cardiometabolic care, with four objectives: (1) to identify the BCTs embedded in these agents, (2) describe how those techniques are delivered, (3) examine the transparency of reporting of LLM design and implementation, and (4) summarize reported user experience and any preliminary behavioral or clinical outcomes. By addressing these objectives, the review seeks to inform the design, evaluation, and reporting of future LLM-driven cardiometabolic interventions and to support clinicians, researchers, policymakers, and regulators in judging whether and how such agents may be integrated into cardiometabolic care pathways.

## Methods

### Overview

This scoping review synthesized studies describing the development, evaluation, or application of LLM-driven conversational agents for cardiometabolic care. The review followed the methodological framework proposed by Arksey and O’Malley [27] and further refined by Levac et al [28], which comprises the following five stages: identifying the research questions, identifying relevant studies, selecting studies, charting and coding the data, and collating, summarizing, and reporting the results. Reporting was guided by the PRISMA-ScR (Preferred Reporting Items for Systematic Reviews and Meta-Analyses extension for Scoping Reviews) checklist [29] (Checklist 1). The protocol, including predefined eligibility criteria, screening procedures, and coding frameworks, was developed a priori and prospectively registered on OSF Registries.

### Research Questions

This review addressed four research questions. First, which BCTs, as defined by the BCTTv1 [22], are embedded in LLM-driven conversational agents designed to support cardiometabolic care? Second, how are these BCTs implemented and delivered through different mechanisms within such agents? Third, how completely and transparently do existing studies report the LLM design and implementation features underpinning these agents, including the underlying model, prompts and system messages, specified roles or personas, handling of conversational context and memory, personalization logic, and safety or oversight mechanisms? Fourth, what has been reported regarding user experience, including acceptability, usability, trust, perceived empathy, engagement, and any preliminary behavioral or clinical outcomes associated with these agents?

### Information Sources and Search Strategy

We conducted a comprehensive literature search in PubMed (MEDLINE), Web of Science Core Collection, Embase, CINAHL, APA PsycInfo, IEEE Xplore, and the ACM Digital Library for the period between January 1, 2020, and November 30, 2025. We also searched arXiv, ClinicalTrials.gov, and the WHO International Clinical Trials Registry Platform. The final search was completed on March 25, 2026. This timeframe was chosen to capture the emergence and early deployment of transformer-based generative language models in health care and to distinguish LLM-driven conversational agents from earlier rule-based or nongenerative systems. All searches were limited to English-language publications. ClinicalTrials.gov and the WHO International Clinical Trials Registry Platform were searched as supplementary identification sources to identify potentially eligible completed or implemented systems and related full-text publications. Registry-only records, protocols, and registrations were not treated as included evidence sources unless a corresponding full-text publication or implemented system report met the eligibility criteria.

The search strategy was developed iteratively by the review team to improve coverage of clinical, behavioral, and interdisciplinary literature, and database-specific queries combined controlled vocabulary and free-text terms related to generative language models (eg, “large language model,” “generative AI,” “ChatGPT,” and “GPT-4”), conversational systems (eg, “conversational agent,” “chatbot,” “virtual coach,” and “dialogue system”), and cardiometabolic conditions (eg, “type 2 diabetes,” “hypertension,” “cardiovascular,” “heart failure,” “cardiometabolic,” and “obesity”). Full search strings for each source are provided in Multimedia Appendix 1.

### Study Selection

Eligibility criteria were as follows: (1) studies published in English; (2) studies describing a patient-facing, text- or voice-based conversational agent that used an LLM or other transformer-based generative foundation model to generate responses during interactions, rather than systems limited to isolated natural language processing components such as entity recognition or risk prediction; (3) studies in which the conversational agent was applied to cardiometabolic prevention, self-management, education, monitoring, or related decision support, including type 2 diabetes, hypertension, cardiovascular disease, heart failure, dyslipidemia, metabolic syndrome, overweight, or obesity explicitly linked to cardiometabolic risk or prevention; and (4) studies that provided sufficient technical detail to confirm or reasonably infer both the use of an LLM or transformer-based generative model and the presence of a patient-facing conversational role in a cardiometabolic context. The inclusion and exclusion criteria are summarized in Table 1.

**Table 1.** Inclusion and exclusion criteria.

Domain	Inclusion criteria	Exclusion criteria
Language and publication status	English-language full-text records published within the prespecified search period	Non-English records, conference abstracts without accessible full text, and inaccessible full-text reports
Clinical scope	Conversational agents addressing cardiometabolic prevention, self-management, education, monitoring, or related decision support	Studies focused primarily on noncardiometabolic conditions
Intervention format	Patient-facing text- or voice-based conversational agents	Nonconversational tools or systems limited to isolated backend functions (eg, entity recognition, risk prediction, or other natural language processing tasks without a patient-facing dialogue role)
AI criterion	Systems using an LLM^a^ or another transformer-based generative model to generate at least part of the conversational output, or systems in which LLM use was reasonably inferable from implementation details such as named tools, prompt-based generation, retrieval-augmented generation, screenshots, generated dialogue examples, or model-specific descriptions	Generic “AI,” “chatbot,” “GenAI^b^,” or “NLP^c^” descriptions without sufficient evidence to verify or reasonably infer LLM use
Study type and evidence source	Empirical studies, formative studies, technical evaluations, and concrete system or prototype papers with a cardiometabolic conversational use case	Reviews, editorials, protocols, conceptual papers, registry-only records, and nonimplemented intervention proposals
Role in care context	Systems with a patient-facing conversational role in a cardiometabolic context	Systems without a patient-facing role or without a relevant cardiometabolic self-management, education, monitoring, or decision-support function

a LLM: large language model.

b GenAI: generative AI.

c NLP: natural language processing.

To classify a system as LLM-driven, we required explicit textual evidence of a named LLM, a transformer-based generative model, an application programming interface linked to such a model, or a sufficiently clear description of RAG or generative dialogue built on a transformer-based foundation model. Reports referring only to “AI,” “chatbot,” “generative artificial intelligence (GenAI),” or “natural language processing (NLP)” in generic terms, without additional supporting evidence of LLM-based generative dialogue, were excluded. For records that described an implemented generative AI (GenAI) conversational agent but did not name a specific model, inclusion required additional supporting evidence, such as explicit LLM or GenAI terminology, prompt-based generation, RAG, prompt-engineering descriptions, screenshots or examples of generated dialogue, or a clearly implemented patient-facing generative component. These cases were retained only when LLM use was judged to be reasonably inferable, and the model description was coded as partially reported or not reported, as appropriate.

Empirical studies, formative studies, and technical or system-level evaluations were eligible if they described a concrete cardiometabolic use case involving direct patient interaction, real users, a clinician, an expert, or other evaluator assessment, or realistic simulated scenarios. We excluded reviews, editorials, conference abstracts without accessible full text, protocols, conceptual papers, registry-only records, and nonimplemented intervention proposals, as well as papers for which the full text could not be obtained.

All records identified through database and registry searches were imported into EndNote (Clarivate) for deduplication and then screened by 2 reviewers (YZ and RG) who independently classified titles and abstracts as include, exclude, or uncertain. Full texts were retrieved for all records classified as including or uncertain and independently assessed against the eligibility criteria. Reasons for exclusion at the full-text stage were recorded. Discrepancies at either stage were resolved through discussion and, when necessary, consultation with a third reviewer (YW). Initial interrater agreement before consensus discussion was calculated for both screening stages. For title and abstract screening, agreement was calculated using the binary decision to retrieve the record for full-text review vs exclude it; initial agreement was 89.8% (Cohen κ=0.78). For full-text eligibility assessment, agreement was calculated using the binary eligible vs ineligible decision; initial agreement was 88.3% (Cohen κ=0.64).

### Data Extraction and Charting

A standardized data extraction form was developed a priori and refined through team discussion. The form was piloted on 3 eligible studies to ensure consistent interpretation of variables and coding rules, with particular attention to BCT identification and delivery mechanism classification. Following this calibration, 2 reviewers (YZ and RG) independently extracted data from all included studies using the standardized form. Any discrepancies were resolved through discussion and, where necessary, consultation with a third reviewer.

Data were charted across the following six prespecified domains: (1) study and population characteristics, including study design, country or region, setting, target conditions, sample size or evaluation unit, participant characteristics, intended end users when different from study participants, intervention duration, and reported behavioral or clinical outcomes; (2) conversational agent and LLM implementation characteristics, including conversational modality, underlying model and access route, role or persona, integration with other AI or rule-based components, handling of conversational context and memory, personalization logic, and any described safety or oversight mechanisms; (3) BCTs, identified and coded according to the BCTTv1 [22]; (4) delivery mechanisms for selected BCTs central to cardiometabolic self-management; (5) user experience and preliminary effectiveness indicators, including usability, acceptability, trust, perceived empathy, engagement, and any reported behavioral or cardiometabolic outcomes; and (6) study classification as empirical human-participant or evaluator-based assessment vs technical, framework-development, simulated-output, proof-of-concept, or other system-level evaluation.

### Coding of BCTs and Delivery Mechanisms

BCTs were identified and coded using the BCTTv1, which specifies 93 hierarchically organized techniques. The full set of 93 BCTs was systematically assessed for each included study. Two reviewers completed formal BCTTv1 training and jointly coded 3 eligible studies to calibrate their interpretation of BCT definitions in the context of LLM-driven conversational interventions. In the main coding phase, included studies were independently assessed for the presence or absence of every BCT, focusing on intervention content that was clearly delivered or intentionally built into the conversational system rather than on generic health information that might be produced opportunistically. A BCT was coded as present only when there was an explicit description or sufficiently clear textual evidence in intervention descriptions, supplementary technical materials, or example dialogues; ambiguous descriptions that could not be confidently mapped to a specific BCT were coded as not present. Discrepancies in BCT coding were resolved through discussion, with a third reviewer consulted if needed. Initial interrater agreement before consensus for study-level BCT presence or absence coding decisions was 88.2%, with a Cohen κ of 0.79. For example, fixed medication reminders delivered through scheduled templates were classified as BCT 7.1 (prompts/cues) at Level 1, whereas personalized lifestyle advice generated in real time from user-entered data was classified as BCT 4.1 (instructions on how to perform behavior) at Level 2.

For a predefined subset of BCTs considered central to cardiometabolic self-management, delivery mechanisms were classified using a study-specific, 3-level framework: Level 0 (static delivery), Level 1 (rule-based or templated delivery), and Level 2 (generative or context-aware delivery). Level 0 denoted invariant or minimally tailored prewritten content; Level 1 denoted content selected from a predefined set of templates according to rules, thresholds, or structured branching logic; and Level 2 denoted content generated in real time by an LLM using user-specific free-text input, conversational history, or rich patient context. When the same BCT appeared to be implemented through more than one mechanism within a study, it was coded as mixed mode rather than forced into a single delivery category. Operational definitions used in eligibility assessment and coding are summarized in Table 2. Because this delivery-level framework was developed for the purposes of this review and has not been formally validated, it should be interpreted as a study-specific analytic classification used to support descriptive synthesis.

**Table 2.** Operational definitions and coding rules used in this review. Delivery-level classification and transparency categories were used as study-specific analytic tools rather than validated measurement instruments.

Term or construct	Operational definition	How it was used in this review
LLM^a^-driven conversational agent	A patient-facing conversational system using a named LLM, another transformer-based generative model, an application programming interface linked to such a model, or a system for which implementation details made LLM-based generative dialogue reasonably inferable	Used to determine eligibility and distinguish included systems from generic AI or chatbot reports without sufficient evidence to verify or reasonably infer LLM use
Patient-facing	Designed for direct interaction with patients or the public through dialogue, including education, coaching, support, monitoring, or guided self-management	Used to exclude backend NLP^b^ tools or nonconversational decision-support systems without a direct dialogue role
Empirical human-participant or evaluator-based study	A study in which patients, intended users, clinicians, experts, or other evaluators constituted the primary evaluated sample and participant-level or evaluator-level outcomes, ratings, or qualitative data were reported.	Used for study classification and MMAT^c^ appraisal when the design matched an MMAT category
Technical or system-level evaluation	An architecture, prototype, benchmark, simulated-dialogue, framework-development, proof-of-concept, or output-evaluation study whose primary focus was system behavior, technical performance, or generated-output assessment rather than participant-level or evaluator-level outcomes. Studies in this category could include limited expert, provider, volunteer, or reviewer assessment of outputs or system operation, but were not treated as MMAT-appraised empirical studies when no analyzable empirical human-participant or evaluator-based study design and outcomes were reported.	Used for study classification and descriptive synthesis; not appraised with MMAT
Confirmed BCT	A BCT^d^ is judged present only when supported by explicit or sufficiently clear textual evidence in the intervention description, additional materials, or example dialogues.	Included in the main synthesis of BCT frequency and distribution
Ambiguous or borderline BCT content	Behavior-related content that could not be mapped confidently to a specific BCT	Not counted as present in the main synthesis
Level 0 delivery	Static or invariant content with no meaningful algorithmic tailoring	Used in delivery-mechanism classification
Level 1 delivery	Rule-based, templated, threshold-based, or branching delivery selected from predefined content	Used in delivery-mechanism classification
Level 2 delivery	Generative or context-aware delivery using real-time LLM output informed by user input, history, or rich patient context	Used in delivery-mechanism classification
Mixed mode	The same BCT is implemented through more than one delivery mechanism within a single study	Reported separately to avoid obscuring hybrid architectures
Fully, partially, or not reported	The degree to which implementation details were explicitly described for each reporting domain	Used in the implementation transparency assessment

a LLM: large language model.

b NLP: natural language processing.

c MMAT: Mixed Methods Appraisal Tool.

d BCT: behavior change technique.

### Quality and Reporting Assessment

Methodological quality was appraised for empirical human-participant or evaluator-based studies using the 2018 version of the Mixed Methods Appraisal Tool (MMAT) [30], which provides design-specific criteria for randomized, nonrandomized, quantitative descriptive, qualitative, and mixed methods studies. Eligible empirical preprints involving human participants, intended users, clinicians, experts, or other evaluators were appraised using the same MMAT procedures. Two reviewers independently rated each applicable MMAT item as “yes,” “no,” or “cannot tell,” and disagreements were resolved through discussion. In line with MMAT guidance, overall summary scores were not calculated; instead, patterns of item-level ratings were used to inform interpretation of the strength and limitations of the empirical evidence base. Technical, prototype, framework-development, proof-of-concept, simulated-output, and other system-level studies without an analyzable empirical human-participant or evaluator-based study design and outcomes were not appraised with MMAT, but were retained to contribute to the mapping of BCTs, delivery mechanisms, and implementation reporting practices. Because these studies were primarily retained to map system functions, delivery mechanisms, and reporting practices rather than to estimate clinical effectiveness, no separate formal technical appraisal framework was retrospectively applied.

To evaluate reporting transparency for LLM implementation, we developed a study-specific structured checklist informed by CONSORT-AI (Consolidated Standards of Reporting Trials–Artificial Intelligence extension) [31], SPIRIT-AI (Standard Protocol Items: Recommendations for Interventional Trials–Artificial Intelligence extension) [32], and existing guidance for digital and behavioral interventions. The checklist covered the following domains considered critical for understanding and reproducing LLM-based conversational systems: description of the underlying model and access route; provision of prompts or representative system messages; specification of the agent’s intended role or persona; handling of conversational context and memory; explanation of personalization logic; reporting of safety guardrails and oversight procedures; and provision of illustrative dialogue examples. Each domain was rated as fully reported, partially reported, or not reported. The checklist was independently applied to all included studies. Discrepancies were resolved through discussion, and domain-level ratings were summarized descriptively to identify common reporting gaps. Initial agreement before consensus for domain-level reporting transparency ratings was 91%, with a Cohen κ of 0.84. Because this checklist was developed for the purposes of this review and was not formally validated, it should be interpreted as a study-specific analytic tool rather than a validated measurement instrument. No study was excluded on the basis of methodological or reporting quality.

### Data Synthesis and Analysis

Data were synthesized using descriptive statistics and narrative synthesis. For the first review question, the frequency and distribution of BCTs were summarized across studies, and the number of studies in which each BCT group and selected individual techniques were identified was reported. For the second review question, BCTs were cross-tabulated by delivery mechanism level and mixed-mode implementation, and, where informative, by study design to explore patterns in how different techniques were implemented through static, rule-based, generative, or hybrid interactions. For the third review question, implementation reporting transparency was summarized descriptively across the predefined checklist domains. MMAT findings were used separately to contextualize the strength and limitations of the empirical human-participant or evaluator-based evidence base rather than as part of the transparency assessment. For the fourth review question, charted data on usability, acceptability, trust, perceived empathy, engagement, and any preliminary behavioral or clinical outcomes were summarized descriptively.

To improve the interpretability of this heterogeneous literature, findings were synthesized separately for empirical human-participant or evaluator-based studies and for technical, framework-development, simulated-output, proof-of-concept, and other system-level studies where appropriate. Additional stratification was undertaken, where informative, by target condition, deployment stage (prototype vs implementation in real-world care), and conversational modality (text vs voice). Studies with limited or poor reporting were retained and explicitly marked as such in the extraction sheet; missing details were coded as “not reported” or “unclear” and were taken into account when interpreting the overall evidence base.

### Ethical Considerations

Ethical approval was not sought for this scoping review because it synthesized data from publicly available published studies and did not involve human participant recruitment, direct participant contact, access to identifiable private information, biological specimens, or individual-level personal health data. In accordance with the Measures for Ethical Review of Life Science and Medical Research Involving Humans [33], ethical review applies to studies involving human participants or the use of human biological specimens or personal information or data; therefore, informed consent, participant privacy procedures, and compensation were not applicable to this review.

## Results

### Study Selection

**Figure 1.**
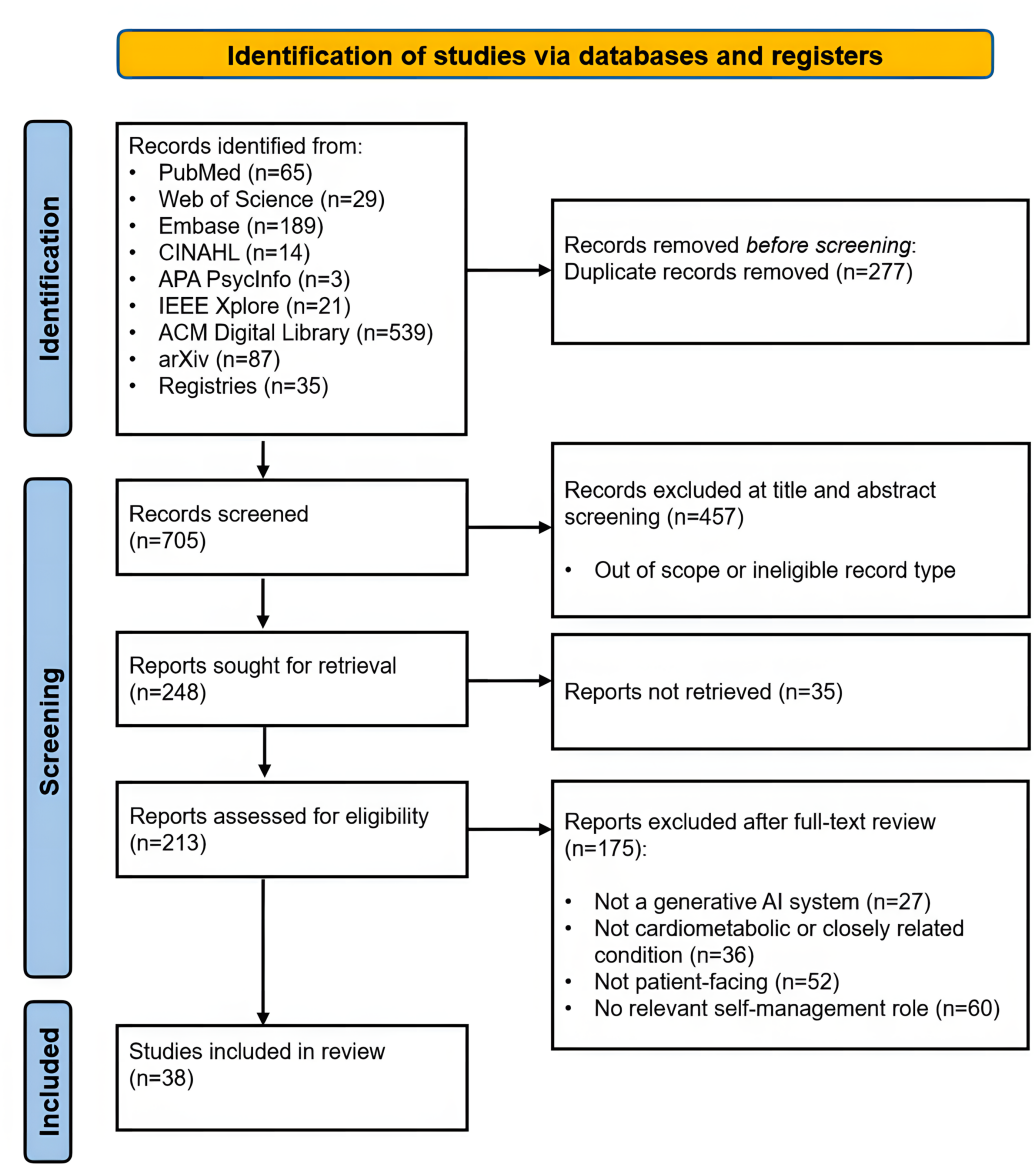
PRISMA (Preferred Reporting Items for Systematic Reviews and Meta-Analyses) flow diagram of study identification, screening, eligibility assessment, and inclusion.

The study selection process is summarized in the PRISMA (Preferred Reporting Items for Systematic Reviews and Meta-Analyses) flow diagram (Figure 1). After removal of duplicates and successive screening of titles and abstracts, followed by full-text assessment, 38 studies met the inclusion criteria and were included in the review. As shown in Figure 1, full-text exclusions were most commonly due to the absence of a relevant self-management role, lack of a patient-facing conversational function, noncardiometabolic focus, or insufficient evidence that the system used a GenAI or LLM architecture.

### Study Characteristics and Methodological Quality

**Figure 2.**
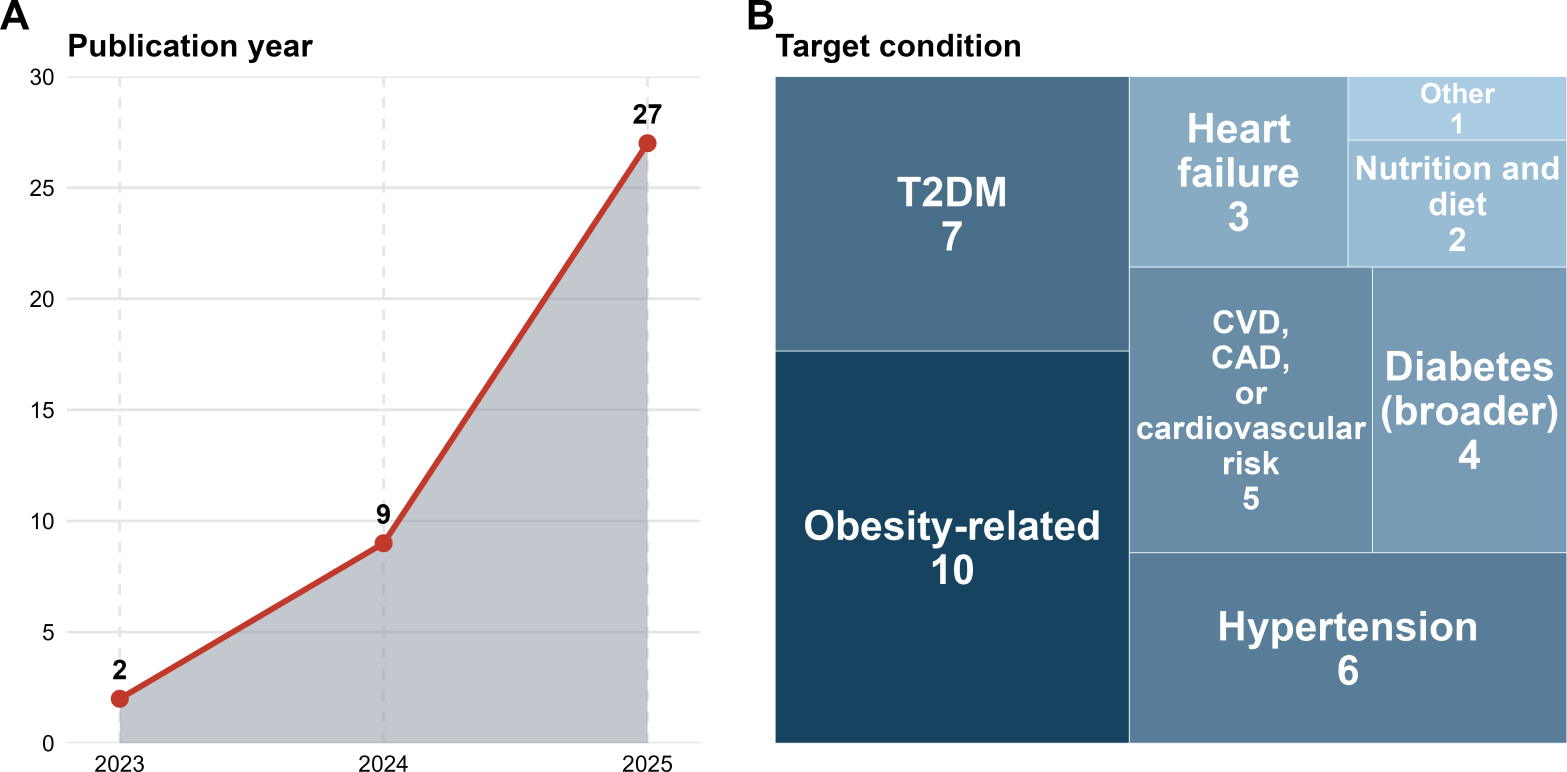
Characteristics of the included studies (n=38). Panel A shows the publication year. Panel B shows the main target condition categories addressed by the included conversational agents. CAD: coronary artery disease; CVD: cardiovascular disease; T2DM: type 2 diabetes mellitus.

Characteristics of the 38 included studies are summarized in Figures 2A and 2B and Table 3. Most included studies were published in 2024‐2025, reflecting a rapidly expanding but still early-stage literature. The included studies came primarily from the United States, Europe, and China, although additional work was identified from Asia, Africa, the Middle East, and multinational collaborations. In design terms, the evidence base remained heterogeneous: 19 studies involved empirical human-participant or evaluator-based assessments, whereas 19 were technical and system-level evaluations, including framework-development, simulated-output, and proof-of-concept studies. Target conditions were most commonly obesity-related conditions (10/38, 26%), followed by type 2 diabetes (7/38, 18%), hypertension (6/38, 16%), cardiovascular disease, coronary artery disease, or cardiovascular risk (5/38, 13%), broader diabetes (4/38, 11%), heart failure (3/38, 8%), nutrition and diet (2/38, 5%), and other multicondition or cross-cutting applications (1/38, 3%).

**Table 3.** Characteristics of included studies (N=38).

Study (first author, year)	Country or region	Condition and population	LLM^a^ model and architecture	Study design	Sample or evaluation unit
Abbasian et al, 2024 [34]	United States	T2DM,^b^ simulated	GPT-3.5-turbo (OpenAI) with RAG^c^	Technical evaluation	100 diabetes-related questions
Aguzzi et al, 2025 [35]	Italy	Hypertension, simulated	Open SLMs^d^ with RAG	Technical evaluation	QA^e^ dataset; 21 evaluation samples
Ahmadi et al, 2025 [36]	United States	Obesity, adults	GPT-4-Turbo	Usability study	25 users
Andreadis et al, 2024 [37]	United States	Hypertension, RPM^f^ patients	GenAI^g^ RPM assistant; model not reported	Formative evaluation	5 clinicians; 5 patients
Antia et al, 2025 [38]	Nigeria	Hypertension, adults	Fine-tuned GPT	Single-arm pilot	50 patients
Cheng et al, 2025 [39]	Taiwan or the United States	Obesity, adults	GPT-3 Davinci with scripts	Randomized controlled trial	97 participants
Chuang et al, 2025 [40]	Taiwan	Multicondition, simulated	ChatGPT API with RAG	Technical evaluation	50 test cases
Coleman et al, 2025 [41]	Ireland	Obesity, adults	Watson Assistant with avatar	Randomized controlled trial	43 participants
Dao et al, 2024 [42]	Ireland or Singapore	Diabetes prevention, simulated	GPT-3.5 with RAG	Technical evaluation	Synthetic profiles and QA prompts
Đurković et al, 2025 [43]	Montenegro	CVD^h^ and arrhythmia, volunteer testing	GPT family with ECG features	Technical proof-of-concept evaluation	Volunteer count NR^i^
Elfayoumi et al, 2025 [44]	Egypt, the United Kingdom, or South Korea	T2DM, simulated	GPT-3.5, Llama, and Gemma with RAG	Technical evaluation	Dataset and model evaluation
Gollapalli and Ng, 2025 [45]	Singapore	T2DM, crowdworker ratings	GPT-4o-mini with RL^j^	Dialogue-system evaluation	717 utterance pairs; 78 snippets
Huang et al, 2025 [46]	United States	Obesity, adults	ChatGPT, GPT-3.5	Experimental survey	87 participants
Hussain and Grundy, 2025 [47]	Australia	Diabetes, simulated	GPT-3.5 and GPT-4	Technical evaluation	Diabetes query scenarios
Jeon et al, 2025 [48]	South Korea	Diabetes, adults	GPT-4 with RAG	Formative evaluation	24 patients; 4 specialists
Kelly et al, 2025 [49]	Ireland	T2DM, simulated	GPT-4o mini with RAG	Technical evaluation	44 curated questions; 16 simulated queries
Kozaily et al, 2023 [50]	United States or Lebanon	Heart failure, simulated patient questions	ChatGPT-3.5 and Bard	Comparative output evaluation	30 HF^k^ questions; repeated queries
Liang et al, 2025 [51]	Hong Kong or the United States	Nutrition, adults	GPT-4	Randomized controlled trial	214 participants
Meng et al, 2025 [52]	China	T2DM, patients	GPT-4, DeepSeek, and Kimi	Mixed methods study	28 participants
Meng et al, 2025 [53]	China	T2DM, patients	GPT-4o with RAG and voice	Nonrandomized trial	40 patients; 20 doctors
Mohd Dan et al, 2025 [54]	Malaysia	Obesity and weight management in adults	ChatGPT o3 with NExGEN prompt generator	Randomized controlled trial	160 participants
Montagna et al, 2023 [55]	Italy	Hypertension, prototype	GPT-3	System design	Prototype and architecture
Mustafa et al, 2025 [56]	Pakistan	T2DM and diabetic retinopathy, adults	Moderated ChatGPT-based QA	Cross-sectional study	51 adults; 137 questions
Neary et al, 2025 [57]	United Kingdom or United States	Obesity, patients	GenAI health coach; model not reported	Framework-development study	5 evaluators; 12 dialogues; patient base NR
Pan et al, 2025 [58]	China	Obesity and metabolic risk, adult	Hunyuan with RAG and multimodal input	Autoethnography	1 participant
Patil et al, 2025 [59]	India	CVD, simulated	BioMistral	Technical evaluation	System testing
Pay et al, 2025 [60]	Turkey	Coronary artery disease, simulated FAQs	ChatGPT-4o, Gemini, and Bing	Comparative output evaluation	50 CAD^l^ FAQs; 2 cardiologists
Ponzo et al, 2024 [61]	Italy	Obesity, simulated cases	Ten general-purpose AI chatbots	Comparative output evaluation	2 cases; 3 dietitians
Rodriguez et al, 2024 [62]	United States	Hypertension, RPM prototype	GPT-4	System prototype	MVP^m^ prototype; no outcome sample
Rossi et al, 2024 [63]	Italy	Diabetes, simulated	WizardLM	Technical diagnostic evaluation	Clinical text dataset
Saraç et al, 2025 [64]	Turkey	Obesity, simulated	GPT-4 and Gemini	Expert-rating study	3 expert trainers
Strömel et al, 2024 [65]	Germany or the European Union	Obesity-related physical activity in adults	GPT-4	Online HCI^n^ experiment	10 interviews; 273 online participants
Szymanski et al, 2024 [66]	United States	Nutrition, dietitians	GPT-4 with RAG	Mixed methods dietitian validation	12 RDs^o^; focus groups
Tayal et al, 2025 [67]	United States	Heart failure, patients	GPT-4 with RAG and voice	Within-subject experiment	20 patients
Tayal et al, 2025 [68]	United States	Heart failure, simulated	GPT-4 and GPT-3.5	Simulation study	Generated HF dialogues
Vats et al, 2025 [69]	India	CAD,^l^ simulated	Gemini and GPT with ML^p^ components	Technical system evaluation	Dataset and model evaluation
Wali et al, 2024 [70]	Canada	CVD risk, simulated	BioMistral with CatBoost	System design	542 cleaned dataset records
Wang et al, 2025 [71]	China	Hypertension, patients	GPT-4o with RAG and agents	Benchmarking and external validation	107 patients; 3 physicians

a LLM: large language model.

b T2DM: type 2 diabetes mellitus.

c RAG: retrieval-augmented generation.

d SLM: small language model.

e QA: question answering.

f RPM: remote patient monitoring.

g GenAI: generative AI.

h CVD: cardiovascular disease.

i NR: not reported.

j RL: reinforcement learning.

k HF: heart failure.

l CAD: coronary artery disease.

m MVP: minimum viable product.

n HCI: human-computer interaction.

o RD: registered dietitian.

p ML: machine learning.

Methodological quality among the 19 empirical human-participant or evaluator-based studies was generally acceptable but heterogeneous (Multimedia Appendix 2). Four studies were randomized, 3 were nonrandomized or external-validation designs, 4 were quantitative descriptive, 1 was qualitative, and 7 used mixed methods designs. Randomized and mixed methods studies were generally rated favorably across MMAT domains. The most common methodological concerns were unclear sampling strategies and uncertain sample representativeness, particularly in quantitative descriptive studies using convenience, online, expert, volunteer, or evaluator samples that did not always match the intended patient end users. In nonrandomized, external-validation, or single-arm studies, causal interpretation was limited by the restricted ability to account for confounding or to attribute observed changes specifically to the conversational agent.

### Behavior Change Content and Delivery Mechanisms

#### BCTs Used

A broad but uneven distribution of BCTs was identified (Table 4; Multimedia Appendix 3). The intervention content was dominated by shaping knowledge and natural consequences: 30 of 38 (79%) studies included instruction on how to perform behavior (BCT 4.1) [34,35,38-42,46-62,64,66-69,71], and 27 of 38 (71%) studies included information about health consequences (BCT 5.1) [34,35,38,40,41,43-45,47-53,56,58-61,63,66-71].

**Table 4.** Frequency and delivery mechanisms of confirmed behavior change techniques identified in included studies (N=38). Mixed mode indicates that the same BCT^a^ within a study, or the same BCT group across techniques within a study, was delivered through more than one mechanism.

BCT^a^ group and label (BCTTv1^b^)	Studies using technique, n (%)	Mixed mode, n	Level 2 only, n^c^	Level 1 only, n^d^	Level 0 only, n^e^
Goals and planning	3 (8)	0	3	0	0
1.1 Goal setting (behavior)	1 (3)	0	1	0	0
1.2 Problem solving	2 (5)	0	2	0	0
1.3 Goal setting (outcome)	1 (3)	0	1	0	0
1.4 Action planning	2 (5)	0	2	0	0
Feedback and monitoring	19 (50)	6	10	3	0
2.2 Feedback on behavior	13 (34)	1	9	3	0
2.3 Self-monitoring of behavior	6 (16)	0	0	6	0
2.4 Self-monitoring of outcomes of behavior	3 (8)	0	0	3	0
2.6 Biofeedback	3 (8)	0	3	0	0
2.7 Feedback on outcome(s) of behavior	4 (11)	1	3	0	0
Social support	8 (21)	0	6	2	0
3.2 Social support (practical)	1 (3)	0	0	1	0
3.3 Social support (emotional)	7 (18)	0	6	1	0
Shaping knowledge	30 (79)	4	23	3	0
4.1 Instruction on how to perform behavior	30 (79)	4	23	3	0
Natural consequences	27 (71)	1	24	2	0
5.1 Information about health consequences	27 (71)	1	24	2	0
Associations	7 (18)	0	3	4	0
7.1 Prompts/cues	7 (18)	0	3	4	0
Repetition and substitution	1 (3)	0	0	1	0
8.1 Behavioral practice/rehearsal	1 (3)	0	0	1	0
Comparison of outcomes	13 (34)	1	11	1	0
9.1 Credible source	13 (34)	1	11	1	0
Regulation	1 (3)	0	1	0	0
11.2 Reduce negative emotions	1 (3)	0	1	0	0
Identity	1 (3)	0	1	0	0
13.2 Framing/reframing	1 (3)	0	1	0	0
Self-belief	1 (3)	1	0	0	0
15.2 Mental rehearsal of successful performance	1 (3)	1	0	0	0

a BCT: behavior change technique.

b BCTTv1: Behavior Change Technique Taxonomy v1.

c Level 2: generative or context-aware delivery.

d Level 1: rule-based, templated, or structured delivery.

e Level 0: static or invariant content.

Feedback and monitoring techniques were also common, appearing in 19 of 38 (50%) studies. The most frequently confirmed techniques in this cluster were feedback on behavior (BCT 2.2; 13/38 studies) [34,36,40-42,48,51,53-55,58,65,67], credible source (BCT 9.1; 13/38 studies) [34,40,43-45,48,49,54,59,60,66,67,71], prompts/cues (BCT 7.1; 7/38 studies) [37,38,42,53,55,62,70], self-monitoring of behavior (BCT 2.3; 6/38 studies) [34,40,53-55,58], feedback on outcomes of behavior (BCT 2.7; 4/38 studies) [44,62,63,70], and self-monitoring of outcomes of behavior (BCT 2.4; 3/38 studies) [53,55,58]. Social support techniques appeared in 8 of 38 (21%) studies, most commonly emotional support (BCT 3.3; 7/38 studies) [39,42,45,46,48,56,71].

In contrast, goals and planning techniques were relatively uncommon, appearing in only 3 of 38 (8%) studies [45,54,64]. More specialized higher-order techniques—such as mental rehearsal of successful performance, behavioral practice/rehearsal, regulation of negative emotions, and framing/reframing—were confined to a small subset of studies [36,45,65]. Because many systems did not fully disclose prompts, dialogue policies, or internal logic, the frequencies reported here should be interpreted as a minimum documented baseline rather than the full functional capability of the systems.

#### Delivery Levels and Hybrid Patterns

**Figure 3.**
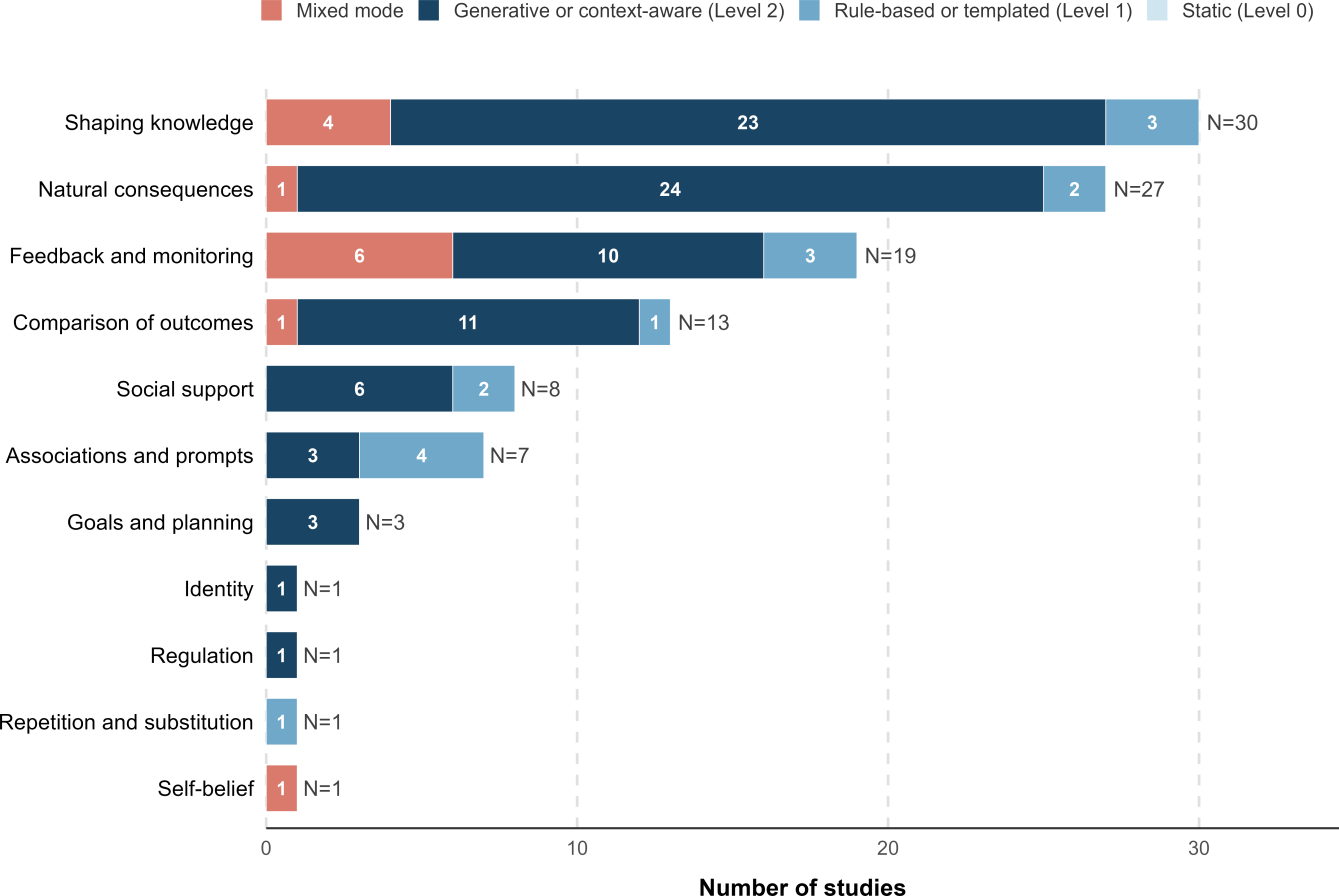
Delivery mechanisms of confirmed behavior change techniques across included studies.

Analysis of delivery mechanisms revealed a distinct functional split between generative explanation and structured data capture (Figure 3; Table 4). For instruction on how to perform behavior (BCT 4.1), 23 of 30 studies used Level 2 delivery, 3 of 30 used Level 1 only, and 4 of 30 used mixed-mode delivery. For information about health consequences (BCT 5.1), 24 of 27 studies used Level 2 delivery, 2 of 27 used Level 1 only, and 1 of 27 used mixed-mode delivery. Emotional social support (BCT 3.3) was largely generative (6/7 Level 2), whereas self-monitoring of behavior and outcomes (BCTs 2.3 and 2.4) remained entirely Level 1 in the confirmed coding. A total of 7 studies exhibited explicit mixed-mode delivery patterns [36,39,43,53,54,67,70]. These hybrid designs typically combined structured or rule-based data entry, reminders, or sensor inputs with LLM-generated explanation, interpretation, or personalized feedback.

### Reported User Experience and Preliminary Behavioral or Clinical Outcomes

User-facing outcomes were heterogeneous and remained focused primarily on proximal rather than long-term clinical endpoints (Multimedia Appendix 4). Across the 19 empirical human-participant or evaluator-based studies, acceptability or satisfaction was reported in 10 studies [36,38,39,41,46,48,51,53,54,67], usability in 5 studies [36,38,41,48,53], trust or credibility in 4 studies [41,48,51,71], and empathy or social support in 6 studies [37,45,46,48,56,71].

Qualitative and narrative feedback frequently suggested that users valued convenience, clarification, and the opportunity to discuss sensitive issues, although perceptions of “synthetic empathy” were mixed [37,48,52,56,58]. Preliminary behavioral or health-status outcomes were reported less consistently: knowledge or self-efficacy outcomes appeared in 4 studies [38,41,53,56], behavioral outcomes in 4 studies [38,45,53,54], and objective physiological or health-status indicators in 3 studies [38,54,58]. Across these studies, positive signals were reported for knowledge gain, engagement, or short-term self-management support, whereas sustained cardiometabolic outcome evidence remained sparse and was usually limited to pilot, short-term, or single-case designs.

### Transparency and Reporting of Implementation Details

Assessment of implementation transparency revealed a systematic reporting imbalance (Table 5; Multimedia Appendix 5). High rates of completeness were observed for model description (33/38, 87%), role or persona (33/38, 87%), personalization logic (27/38, 71%), and example dialogues (32/38, 84%). By contrast, only 13 of 38 (34%) studies fully reported their prompts or system messages [35,39,42,45,49,50,60,61,64-68], while 18 of 38 (47%) studies provided only partial prompt information.

**Table 5.** Completeness of reporting for key LLM^a^ implementation features across included studies (N=38).

Reporting domain	Item description	Fully reported, n (%)^b^	Partially reported, n (%)^c^	Not reported, n (%)^d^
Model transparency	Specific LLM architecture and version	33 (87)	5 (13)	0 (0)
System prompts	Exact phrasing of system instructions or prompt templates	13 (34)	18 (47)	7 (18)
Agent persona	Defined role or persona assigned to the agent	33 (87)	1 (3)	4 (11)
Context and memory	Handling of conversational history or RAG^e^ context	21 (55)	0 (0)	17 (45)
Personalization	Logic for tailoring output to user data or profiles	27 (71)	3 (8)	8 (21)
Safety mechanisms	Guardrails, refusal rules, or human oversight procedures	16 (42)	7 (18)	15 (39)
Dialogue examples	Verbatim examples of LLM-generated dialogue	32 (84)	0 (0)	6 (16)

a LLM: large language model.

b Fully reported indicates that the item was explicitly described with sufficient detail for replication.

c Partially reported indicates that the item was mentioned or described conceptually but lacking specific implementation details.

d Not reported indicates that the item was absent or not described.

e RAG: retrieval-augmented generation.

Safety or oversight mechanisms were fully reported in 16 of 38 (42%) studies [39-41,43,48,49,51,55-58,62,65-67,71], partially reported in 7 of 38 (18%) studies [34,42,52,54,61,64,70], and not reported in the remaining 15 of 38 (39%) studies. Context and memory handling were clearly described in 21 of 38 (55%) studies [34,36,39-42,45,48-51,53,54,57,58,61-63,67,70,71], but were not reported in 17 of 38 (45%) studies. Incomplete reporting of prompts, context handling, and safety procedures also limits confidence that the documented BCT profile fully captures system behavior.

### Conceptual Synthesis of the LLM-Behavior Change Pipeline

**Figure 4.**
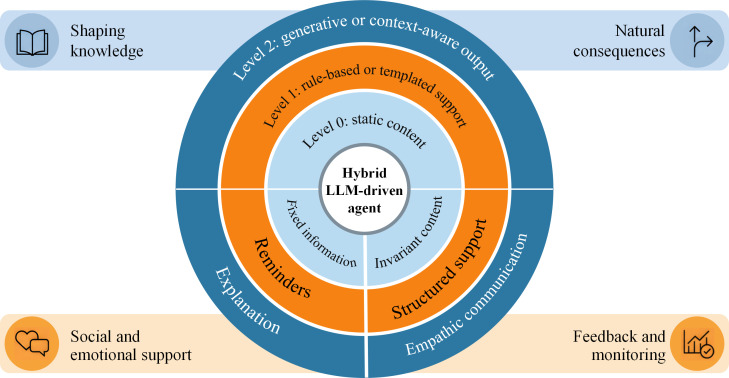
Hybrid delivery and behavior change support in cardiometabolic conversational agents.

Figure 4 summarizes how LLM-driven agents for cardiometabolic care connect technical design with behavior change. User and clinical data, together with guidelines and other knowledge sources (often via RAG), feed into a three-level delivery stack: static content (Level 0), rule-based or templated messages (Level 1), and generative, context-aware LLM outputs (Level 2), which are frequently combined in mixed-mode delivery. These mechanisms implement clusters of BCTs—particularly shaping knowledge, natural consequences, feedback and monitoring, and social or emotional support—and are wrapped by reporting, safety, and oversight processes. Across the current literature, evaluation remains concentrated in feasibility, acceptability, framework-development, simulated-output, proof-of-concept, and technical-assessment settings rather than in long-term comparative outcome studies.

## Discussion

### Principal Findings

This scoping review mapped 38 studies of LLM-driven conversational agents for cardiometabolic care across a spectrum from technical and system-level evaluations, framework-development studies, simulated-output studies, and proof-of-concept reports to empirical human-participant or evaluator-based assessments, small pilot trials, and randomized evaluations. Most agents were positioned primarily as educators or coaches, focusing on explanation, data interpretation, and question answering, whereas only a smaller subset explicitly defined sustained behavior change as a primary outcome.

When coded using the BCTTv1, the most frequently implemented techniques belonged to the shaping knowledge, natural consequences, and feedback and monitoring clusters, with techniques from identity, self-belief, comparison of behavior, antecedents, and repetition and substitution rarely reported. Generative LLM outputs were mainly used to deliver knowledge-related techniques and socioemotional responses, whereas prompts, reminders, and structured self-monitoring remained largely rule-based or templated. Reporting of implementation details was uneven: most studies identified the underlying model and agent role, but relatively few described prompts, handling of conversational context and memory, or explicit safety guardrails and escalation pathways in sufficient detail. Empirical evaluations remained limited, with 19 studies involving empirical human-participant or evaluator-based assessments and 19 classified as technical, framework-development, simulated-output, proof-of-concept, or other system-level evaluations. Reported outcomes were concentrated on usability, perceived helpfulness, and other proximal indicators rather than on sustained behavioral or clinical endpoints, suggesting that this literature is best understood as an early-stage, rapidly evolving design space rather than a mature body of effectiveness evidence for cardiometabolic outcomes [10,72,73].

### Behavior Change Content and Focus of Current Systems

The BCT coding highlights a clear emphasis on educational and informational functions. Taken together, this pattern suggests that current LLM-driven agents are being used primarily as educational and interpretive interfaces rather than as fully developed behavior-change programs, a profile consistent with the long-standing dominance of information provision and self-monitoring in digital cardiometabolic self-management tools [74-77]. In sensor-integrated systems, LLMs were often used to translate complex data streams, including glucose, blood pressure, or activity patterns, into narrative feedback that highlighted trends, discrepancies between current and desired states, or reframed fluctuations in a neutral or supportive tone. Such uses suggest a potential to extend traditional educational functions into more reflective engagement with personal data, although direct comparative evidence remains limited.

By contrast, comparatively few systems implemented more demanding techniques such as collaborative goal setting, detailed action planning, structured problem solving, identity work, or deliberate strengthening of self-efficacy and self-belief, and when they did, these components were usually briefly described and rarely evaluated rigorously. Social support was typically expressed as general empathy, reassurance, and normalizing statements rather than through structured social processes, such as involving family members or peers or explicitly addressing social determinants of health [10,24]. Overall, most agents operate primarily as enhanced educators and explainers rather than as comprehensive behavioral coaches capable of working systematically with motivation, habits, identity, and social context over time. Viewed through a behavior change lens, current LLM-enabled agents remain largely reactive: they respond to questions, readings, or simple screening inputs but seldom orchestrate proactive, multisession behavior change trajectories. This pattern underscores the gap between current informational use and more comprehensive behavior-change support for long-term cardiometabolic risk reduction.

### Delivery Mechanisms and the Role of Hybrid Architectures

By classifying BCT delivery mechanisms into static (Level 0), rule-based or templated (Level 1), and generative or context-aware (Level 2), this review suggests that LLMs are typically embedded within hybrid architectures rather than replacing existing logic entirely [78,79]. In practice, generative delivery was concentrated in explanatory, interpretive, and socioemotional functions, whereas prompts, structured self-monitoring, and some safety-sensitive processes remained more likely to rely on deterministic rules or templates [80-86]. Mixed-mode systems, therefore, appeared to divide labor pragmatically: rule-based components captured data, triggered alerts, or bounded risk, while LLMs translated these outputs into personalized narratives or supportive explanations [87-90].

This pattern suggests a pragmatic division of labor, in which contemporary LLMs, still error-prone for precise numerical reasoning and threshold logic in safety-critical settings, are used to translate structured outputs into lay explanations and to integrate data, guidance, and psychosocial support into coherent narratives, while conventional interfaces, sensors, and deterministic rules handle tightly bounded decisions [91-94]. At the same time, hybrid systems can blur boundaries for end users, who typically perceive a single conversational agent rather than a composition of modules; without clear communication of which outputs arise from validated rules and which from probabilistic generative models, there is a risk that users overattribute reliability and authority to the conversational layer [95-98]. Future implementations should therefore not only refine internal orchestration but also consider how to make these internal boundaries and confidence levels transparent and meaningful to patients and clinicians [80,99].

### Reporting Transparency, Prompts, and Safety

The reporting framework applied in this review identified substantial gaps in transparency around key implementation features. Although most studies named the underlying model or vendor and described the agent’s intended role, detailed reporting of prompts, context handling, memory, and safety architecture remained inconsistent. Personalization logic was often summarized only in generic terms, leaving unclear which user variables were available to the model and how they influenced generation. In a small number of studies, the system was described as GenAI- or LLM-enabled but the specific model architecture or version was not reported, requiring classification based on reasonable inference from implementation details.

These reporting gaps make it difficult to anticipate how systems would behave outside the study context, to assess alignment with emerging AI reporting guidance [31,32,100], or to adapt interventions to other populations and health care systems. They also constrain the ability to link specific BCTs to concrete implementation choices, such as prompt design, configuration of RAG, or escalation rules. Incomplete reporting of prompts, dialogue policies, and context handling also means that the observed BCT profile is likely to represent a minimum documented baseline rather than the full functional capability of these systems. Conceptually, prompts, retrieval configurations, and dialogue policies constitute active components of the intervention [101-103]. In the context of pharmacological trials, it would be unacceptable to evaluate a drug without disclosing the active compound and dose; by analogy, in LLM-based interventions, failure to report core system instructions, retrieval scope, and safety guardrails effectively renders the intervention a black box [104,105]. As cardiometabolic conversational agents move closer to deployment, more consistent and granular reporting of these elements will be essential for replication, critical appraisal, and responsible reuse [15].

### Methodological Quality, Clinical Readiness, and Evidence Gaps

The MMAT-based appraisal and overall distribution of study designs indicate that the evidence base remains preliminary and methodologically uneven. Among the 19 empirical human-participant or evaluator-based studies, randomized studies were few and were generally short, modest in size, and oriented toward proximal outcomes such as knowledge, intentions, usability, or satisfaction rather than cardiometabolic endpoints. Common methodological concerns included unclear sampling strategies, uncertain sample representativeness, and limited ability in nonrandomized or single-arm designs to account for confounding or attribute observed changes specifically to the conversational agent. Qualitative and mixed methods studies were more informative about acceptability, perceived empathy, and implementation barriers than about clinical impact, whereas technical, framework-development, simulated-output, proof-of-concept, and other system-level studies primarily illuminated system architecture, retrieval performance, or possible failure modes rather than real-world behavior change or health outcomes [9,106-110]. Sustained engagement beyond a few weeks, longer-term adherence, and effects on cardiometabolic risk markers therefore remain largely unexplored.

Safety considerations further constrain conclusions about clinical readiness. Several evaluation-focused papers documented instances of inaccurate or incomplete advice, overconfident recommendations, and context gaps, particularly in higher-risk situations such as insulin dosing, severe hyperglycemia, or acute cardiovascular symptoms [19,111,112]. However, fewer than half of the studies clearly described guardrails, refusal rules, or human oversight procedures, and almost none reported systematic monitoring of adverse events or near misses. In this context, high levels of user trust and positive responses to “synthetic empathy” may increase risk if they are not matched by robust safeguards and clear communication about limitations [113,114]. Overall, the current literature is sufficient to characterize design patterns, BCT content, and early user reactions, but not to support strong conclusions about effectiveness or safety in long-term cardiometabolic management. There is a particular lack of evidence on sustained engagement, unintended consequences—such as overreliance on AI advice or erosion of trust in clinicians—and differential impacts across sociodemographic groups, languages, and health literacy levels [115,116].

### Implications for Design and Practice

Several implications for the design and deployment of future cardiometabolic conversational agents emerge from this synthesis.

First, the strong emphasis on educational BCTs suggests an opportunity to broaden the behavioral repertoire of LLM-driven agents. Designers could more deliberately incorporate techniques related to collaborative goal setting, graded action planning, problem solving, coping planning, and strengthening self-efficacy, building on established behavior change theories [76,117] rather than relying predominantly on information provision and reassurance. The flexibility of generative models, when combined with structured dialogue frameworks and explicit state representations, may be well suited to iterative negotiation and refinement of goals, yet this potential remains largely unexplored [118].

Second, the observed hybrid architectures highlight the importance of explicitly deciding which functions should remain rule-based and which should be delegated to generative models. For high-stakes decisions, deterministic logic and validated clinical rules are likely to remain essential, with LLMs used to provide explanatory narratives, motivational framing, and synthetic empathy [85,86]. For more discretionary functions, such as narrative reflection on data, context-sensitive lifestyle suggestions, or supportive conversations about stress and motivation, generative models may add value beyond traditional templates. Making these design choices explicit could improve both safety and interpretability and may facilitate clearer communication with regulators and clinical stakeholders.

Third, the persistent gaps in reporting suggest that teams developing LLM-based cardiometabolic agents should treat transparency as a core design requirement. Summarizing prompt templates, describing how user data and context are provided to the model, documenting safety guardrails and human oversight workflows [119], and presenting representative interaction transcripts can support critical appraisal, replication, and responsible adaptation, and are necessary for governance decisions regarding risk assessment, consent processes, integration with clinical pathways, and alignment with regulatory expectations [120,121].

Finally, early evaluations focusing on usability and perceived empathy, although informative, should not substitute for studies that examine integration into real care pathways [122]. Evaluations that embed LLM-driven agents alongside clinicians, remote monitoring programs, or existing digital platforms will be central to understanding how these systems affect workload, communication patterns, equity, trust, and patient outcomes; in practice, generative agents are likely to be most useful as supportive layers within multidisciplinary, multicomponent interventions rather than as stand-alone solutions [123].

### Implications for Future Research

Future research should move beyond framework-development, simulated-output, proof-of-concept, and short-term usability studies toward more rigorous evaluations of clinical and behavioral impact. Priority directions include adequately powered randomized or quasi-experimental trials comparing LLM-enhanced interventions with best-available digital or human-delivered care, with follow-up long enough to capture meaningful changes in glycemic control, blood pressure, weight, physical activity, and other cardiometabolic outcomes [122]. Study designs that isolate the added value of generative components relative to template-based chatbots or purely rule-based decision support would be particularly informative. There is also a need for more systematic assessment of potential harms and unintended effects. Studies should monitor inaccurate or unsafe advice, overconfidence in recommendations, confusion about the agent’s role, overreassurance or under-escalation, and interactions with existing health beliefs and care relationships [21,95,124].

From a behavioral science perspective, future work could experimentally manipulate BCT content and delivery mechanisms within otherwise similar LLM-based systems to test which combinations of techniques and implementation levels produce robust and equitable behavior change. Mixed methods approaches that integrate fine-grained log data, conversation analyses, and in-depth interviews may deepen understanding of how users engage with generative agents over time, how they interpret “synthetic empathy,” and how feedback loops around self-monitoring and narrative reflection operate in routine use [125]. In parallel, methodological and reporting standards tailored to LLM-based conversational interventions should be further developed and adopted. Building on existing AI trial extensions and digital health reporting frameworks, such standards could specify minimum requirements for describing models, prompts, training data, retrieval processes, safety guardrails, and human oversight arrangements, as well as expectations for sharing code, configuration files, or synthetic prompts where feasible. Agreement on such standards would substantially improve the interpretability, comparability, and cumulative value of future cardiometabolic trials involving GenAI [21,126].

### Limitations

This review has several limitations that should be acknowledged. First, as a scoping rather than a systematic review with meta-analysis, the focus was on breadth and mapping rather than pooled estimates of effectiveness. Although empirical human-participant or evaluator-based studies were appraised with the MMAT, the review was not designed to produce an overall certainty-of-evidence rating or quantitative effect estimate. Second, inclusion was restricted to English-language publications and English-language databases within a defined time window, which may have underrepresented work from non-English-speaking regions and model ecosystems; accordingly, conclusions about the global landscape should be interpreted with caution.

Third, BCT and delivery-level coding depended on the quality of reporting; in many cases, sparse descriptions may have led to underestimation of the complexity of implemented techniques or the extent of generative delivery, particularly where prompts and internal logic were not disclosed. The observed BCT profile should therefore be interpreted as a minimum documented baseline rather than a complete representation of system capability.

Fourth, a small number of systems were retained because LLM use was reasonably inferable from GenAI terminology, prompt-based generation, RAG, prompt-engineering descriptions, screenshots or examples of generated dialogue, or implemented patient-facing generative functions, although the specific model architecture or version was not explicitly reported. These cases introduced some uncertainty in classifying systems as LLM-driven and were coded as partially reported for model transparency.

Fifth, the field is evolving extremely rapidly, and newer models, architectures, and governance approaches may not yet be represented in the published literature. Sixth, both the delivery-level framework and the implementation transparency checklist were review-specific analytic tools informed by existing guidance rather than formally validated measurement instruments. Seventh, although we coded granular BCT content, we did not extract whether interventions were explicitly grounded in higher-level behavioral theories. Eighth, technical, framework-development, simulated-output, proof-of-concept, and other system-level studies were retained to map design and implementation patterns, but no separate formal technical appraisal framework was applied to those records. Finally, because many included studies lacked behavioral or clinical outcomes, the synthesis necessarily centers on design features and early-stage evaluations rather than on hard health impact, and conclusions regarding effectiveness and safety must remain tentative.

### Conclusions

LLM-driven conversational agents for cardiometabolic care are proliferating and increasingly sophisticated. Across the studies identified in this review, generative models were used predominantly as educational and explanatory tools, and as vehicles for narrative feedback and “synthetic empathy,” whereas more complex and longitudinal BCTs remained relatively uncommon. Generative components are most often deployed to shape knowledge, interpret data, and convey emotional support, while prompts, reminders, self-monitoring pathways, and safety-critical functions remain largely rule-based or static within hybrid architectures. Reporting of implementation details, personalization logic, and safety guardrails is inconsistent, and empirical human-participant or evaluator-based evaluations are mostly small, short-term, and focused on usability or perceived quality rather than on sustained behavior change or clinical outcomes. The current evidence base is therefore most useful for identifying design patterns and reporting gaps, rather than for drawing firm conclusions about long-term clinical effectiveness.

## Supplementary material

10.2196/89190 Multimedia Appendix 1 Full database and registry search strategies.

10.2196/89190 Multimedia Appendix 2 Mixed Methods Appraisal Tool assessment of empirical human-participant or evaluator-based studies.

10.2196/89190 Multimedia Appendix 3 Detailed mapping of confirmed behavior change techniques and delivery-level classifications.

10.2196/89190 Multimedia Appendix 4 Detailed characteristics of interventions, comparators, duration, and reported outcomes.

10.2196/89190 Multimedia Appendix 5 Detailed assessment of implementation-reporting transparency for large language model–driven conversational agents.

10.2196/89190 Checklist 1 PRISMA-ScR checklist.

## Abbreviations

BCT: behavior change technique
BCTTv1: Behavior Change Technique Taxonomy v1
CONSORT-AI: Consolidated Standards of Reporting Trials–Artificial Intelligence extension
GenAI: generative AI
LLM: large language model
MMAT: Mixed Methods Appraisal Tool
PRISMA: Preferred Reporting Items for Systematic Reviews and Meta-Analyses
PRISMA-ScR: Preferred Reporting Items for Systematic Reviews and Meta-Analyses extension for Scoping Reviews
RAG: retrieval-augmented generation
SPIRIT-AI: Standard Protocol Items: Recommendations for Interventional Trials–Artificial Intelligence extension
