# Supplementary material for: Behavior Change Content and Implementation of Large Language Model–Driven Conversational Agents in Cardiometabolic Care: Scoping Review
Source: J Med Internet Res. 2026 Jul 15;28:e89190. doi: 10.2196/89190 (PMC13372078; doi:10.2196/89190)
Supplement: Multimedia Appendix 1 [file jmir-v28-e89190-s001.docx]

**Multimedia Appendix 1: Full database and registry search strategies**

Searches were conducted in PubMed (MEDLINE), Web of Science Core Collection, Embase, CINAHL, APA PsycInfo, IEEE Xplore, the ACM Digital Library, arXiv, ClinicalTrials.gov, and the WHO International Clinical Trials Registry Platform (ICTRP). All searches covered records published between January 1, 2020 and November 30, 2025, were limited to English-language publications unless otherwise noted, and were finalized on March 25, 2026.

ClinicalTrials.gov and the WHO ICTRP were searched as supplementary identification sources. Registry-only records, protocols, and registrations were used only to identify potentially eligible completed or implemented systems and related full-text reports, and were not treated as included evidence sources unless a corresponding full-text publication or implemented system report met the eligibility criteria.

# **PubMed (MEDLINE)**

The following search strategy was used in PubMed (MEDLINE). The search was run on March 25, 2026 and was limited to articles published between January 1, 2020 and November 30, 2025, and to English-language publications.

("large language model"[tiab] OR "large language models"[tiab] OR "large-language model"[tiab] OR "large-language models"[tiab]
 OR "generative AI"[tiab] OR "generative artificial intelligence"[tiab]
 OR "Generative Pre-trained Transformer"[tiab]
 OR "foundation model"[tiab] OR "foundation models"[tiab]
 OR ChatGPT[tiab] OR "GPT-4"[tiab] OR "GPT-3.5"[tiab] OR "GPT-3"[tiab]
 OR gpt4[tiab] OR gpt3[tiab] OR gpt3.5[tiab]
 OR LLaMA[tiab] OR "Llama 2"[tiab]
 OR Claude[tiab] OR Gemini[tiab] OR Bard[tiab] OR deepseek[tiab])
AND
("conversational agent"[tiab] OR "conversational agents"[tiab] OR "conversational AI"[tiab]
 OR chatbot[tiab] OR chatbots[tiab] OR "chat bot"[tiab]
 OR "dialogue system"[tiab] OR "dialogue systems"[tiab]
 OR "virtual agent"[tiab] OR "virtual agents"[tiab]
 OR "virtual coach"[tiab] OR "virtual assistant"[tiab] OR "virtual nurse"[tiab])
AND
("Diabetes Mellitus, Type 2"[Mesh] OR "diabetes mellitus, type 2"[tiab] OR "type 2 diabetes"[tiab] OR T2DM[tiab]
 OR "Hypertension"[Mesh] OR hypertension[tiab] OR "high blood pressure"[tiab]
 OR "Heart Failure"[Mesh] OR "heart failure"[tiab]
 OR "Cardiovascular Diseases"[Mesh] OR "cardiovascular disease"[tiab] OR "cardiovascular diseases"[tiab]
 OR "coronary heart disease"[tiab] OR "ischemic heart disease"[tiab]
 OR "Obesity"[Mesh] OR obes*[tiab]
 OR "Metabolic Syndrome"[Mesh] OR "metabolic syndrome"[tiab]
 OR "Overweight"[Mesh] OR overweight[tiab]
 OR "Dyslipidemias"[Mesh] OR dyslipidemia[tiab] OR dyslipidemias[tiab]
 OR hyperlipidemia[tiab] OR hyperlipidemias[tiab] OR cholesterol[tiab])
AND ("2020/01/01"[dp] : "2025/11/30"[dp])
AND english[lang]

Records retrieved before deduplication: 65.

# **Web of Science Core Collection**

The following topic (TS) search was used in Web of Science Core Collection. The search was run on March 25, 2026, with filters applied for English language and publication years 2020–2025.

TS=(
 ("large language model*" OR "generative AI" OR "generative artificial intelligence"
 OR "Generative Pre-trained Transformer" OR "foundation model*"
 OR ChatGPT OR "GPT-4" OR "GPT-3.5"
 OR Llama OR LLaMA OR Claude OR Gemini OR Bard OR deepseek
 OR "large generative model*")
 AND
 ("conversational agent*" OR "conversational AI" OR chatbot* OR "chat bot*"
 OR "dialogue system*" OR "dialog system*" OR "conversational system*"
 OR "virtual agent*" OR "virtual coach" OR "virtual assistant")
 AND
 (cardiometabolic OR "type 2 diabetes" OR "diabetes mellitus type 2" OR T2DM
 OR hypertension OR "cardiovascular disease" OR "cardiovascular diseases"
 OR "coronary heart disease" OR "ischemic heart disease" OR "heart failure"
 OR obes* OR "metabolic syndrome" OR overweight OR dyslipidemia OR dyslipidemias
 OR hyperlipidemia OR hyperlipidemias OR cholesterol)
)

Refined by: LANGUAGE: (English); PUBLICATION YEARS: (2020–2025).

Records retrieved before deduplication: 29.

# **Embase**

The following search strategy was used in Embase. The search was run on March 25, 2026, with publication-date and language limits aligned with the overall review strategy.

(
 'large language model':ti,ab,kw OR 'large language models':ti,ab,kw
 OR 'large-language model':ti,ab,kw OR 'large-language models':ti,ab,kw
 OR 'generative ai':ti,ab,kw OR 'generative artificial intelligence':ti,ab,kw
 OR 'generative pre-trained transformer':ti,ab,kw
 OR 'foundation model':ti,ab,kw OR 'foundation models':ti,ab,kw
 OR chatgpt:ti,ab,kw OR 'gpt-4':ti,ab,kw OR 'gpt-3.5':ti,ab,kw OR 'gpt-3':ti,ab,kw
 OR gpt4:ti,ab,kw OR gpt3:ti,ab,kw OR gpt3.5:ti,ab,kw
 OR llama:ti,ab,kw OR 'llama 2':ti,ab,kw
 OR claude:ti,ab,kw OR gemini:ti,ab,kw OR bard:ti,ab,kw OR deepseek:ti,ab,kw
)
AND
(
 'conversational agent':ti,ab,kw OR 'conversational agents':ti,ab,kw
 OR 'conversational ai':ti,ab,kw
 OR chatbot:ti,ab,kw OR chatbots:ti,ab,kw OR 'chat bot':ti,ab,kw
 OR 'dialogue system':ti,ab,kw OR 'dialogue systems':ti,ab,kw
 OR 'virtual agent':ti,ab,kw OR 'virtual agents':ti,ab,kw
 OR 'virtual coach':ti,ab,kw OR 'virtual assistant':ti,ab,kw OR 'virtual nurse':ti,ab,kw
)
AND
(
 'type 2 diabetes mellitus'/exp OR 'type 2 diabetes mellitus':ti,ab,kw
 OR 'type 2 diabetes':ti,ab,kw OR t2dm:ti,ab,kw
 OR 'hypertension'/exp OR hypertension:ti,ab,kw OR 'high blood pressure':ti,ab,kw
 OR 'heart failure'/exp OR 'heart failure':ti,ab,kw
 OR 'cardiovascular disease'/exp OR 'cardiovascular disease':ti,ab,kw
 OR 'cardiovascular diseases':ti,ab,kw
 OR 'coronary heart disease':ti,ab,kw OR 'ischemic heart disease':ti,ab,kw
 OR 'obesity'/exp OR obes*:ti,ab,kw
 OR 'metabolic syndrome'/exp OR 'metabolic syndrome':ti,ab,kw
 OR 'overweight'/exp OR overweight:ti,ab,kw
 OR 'dyslipidemia'/exp OR dyslipidemia:ti,ab,kw OR dyslipidemias:ti,ab,kw
 OR hyperlipidemia:ti,ab,kw OR hyperlipidemias:ti,ab,kw
 OR cholesterol:ti,ab,kw
)
AND [2020-2025]/py
AND [english]/lim

Records retrieved before deduplication: 189.

# **CINAHL**

The following search strategy was used in CINAHL via EBSCOhost. The search was run on March 25, 2026, with publication-date and language limits aligned with the overall review strategy.

(
 TI (
 "large language model" OR "large language models" OR "large-language model" OR "large-language models"
 OR "generative AI" OR "generative artificial intelligence"
 OR "Generative Pre-trained Transformer"
 OR "foundation model" OR "foundation models"
 OR ChatGPT OR "GPT-4" OR "GPT-3.5" OR "GPT-3"
 OR gpt4 OR gpt3 OR gpt3.5
 OR LLaMA OR "Llama 2" OR Claude OR Gemini OR Bard OR deepseek
 )
 OR
 AB (
 "large language model" OR "large language models" OR "large-language model" OR "large-language models"
 OR "generative AI" OR "generative artificial intelligence"
 OR "Generative Pre-trained Transformer"
 OR "foundation model" OR "foundation models"
 OR ChatGPT OR "GPT-4" OR "GPT-3.5" OR "GPT-3"
 OR gpt4 OR gpt3 OR gpt3.5
 OR LLaMA OR "Llama 2" OR Claude OR Gemini OR Bard OR deepseek
 )
)
AND
(
 TI (
 "conversational agent" OR "conversational agents" OR "conversational AI"
 OR chatbot OR chatbots OR "chat bot"
 OR "dialogue system" OR "dialogue systems"
 OR "virtual agent" OR "virtual agents"
 OR "virtual coach" OR "virtual assistant" OR "virtual nurse"
 )
 OR
 AB (
 "conversational agent" OR "conversational agents" OR "conversational AI"
 OR chatbot OR chatbots OR "chat bot"
 OR "dialogue system" OR "dialogue systems"
 OR "virtual agent" OR "virtual agents"
 OR "virtual coach" OR "virtual assistant" OR "virtual nurse"
 )
)
AND
(
 MH "Diabetes Mellitus, Type 2+"
 OR MH "Hypertension+"
 OR MH "Heart Failure+"
 OR MH "Cardiovascular Diseases+"
 OR MH "Obesity+"
 OR MH "Metabolic Syndrome+"
 OR MH "Overweight+"
 OR
 TI (
 cardiometabolic OR "diabetes mellitus, type 2" OR "type 2 diabetes" OR T2DM
 OR hypertension OR "high blood pressure"
 OR "heart failure"
 OR "cardiovascular disease" OR "cardiovascular diseases"
 OR "coronary heart disease" OR "ischemic heart disease"
 OR obes* OR "metabolic syndrome" OR overweight
 OR dyslipidemia OR dyslipidemias OR hyperlipidemia OR hyperlipidemias
 OR cholesterol
 )
 OR
 AB (
 cardiometabolic OR "diabetes mellitus, type 2" OR "type 2 diabetes" OR T2DM
 OR hypertension OR "high blood pressure"
 OR "heart failure"
 OR "cardiovascular disease" OR "cardiovascular diseases"
 OR "coronary heart disease" OR "ischemic heart disease"
 OR obes* OR "metabolic syndrome" OR overweight
 OR dyslipidemia OR dyslipidemias OR hyperlipidemia OR hyperlipidemias
 OR cholesterol
 )
)

Limiters applied: English Language; Published Date: 20200101–20251130.

Records retrieved before deduplication: 14.

# **APA PsycInfo**

The following search strategy was used in APA PsycInfo via EBSCOhost. The search was run on March 25, 2026, with publication-date and language limits aligned with the overall review strategy.

(
 TI (
 "large language model" OR "large language models" OR "large-language model" OR "large-language models"
 OR "generative AI" OR "generative artificial intelligence"
 OR "Generative Pre-trained Transformer"
 OR "foundation model" OR "foundation models"
 OR ChatGPT OR "GPT-4" OR "GPT-3.5" OR "GPT-3"
 OR gpt4 OR gpt3 OR gpt3.5
 OR LLaMA OR "Llama 2" OR Claude OR Gemini OR Bard OR deepseek
 )
 OR
 AB (
 "large language model" OR "large language models" OR "large-language model" OR "large-language models"
 OR "generative AI" OR "generative artificial intelligence"
 OR "Generative Pre-trained Transformer"
 OR "foundation model" OR "foundation models"
 OR ChatGPT OR "GPT-4" OR "GPT-3.5" OR "GPT-3"
 OR gpt4 OR gpt3 OR gpt3.5
 OR LLaMA OR "Llama 2" OR Claude OR Gemini OR Bard OR deepseek
 )
)
AND
(
 TI (
 "conversational agent" OR "conversational agents" OR "conversational AI"
 OR chatbot OR chatbots OR "chat bot"
 OR "dialogue system" OR "dialogue systems"
 OR "virtual agent" OR "virtual agents"
 OR "virtual coach" OR "virtual assistant" OR "virtual nurse"
 )
 OR
 AB (
 "conversational agent" OR "conversational agents" OR "conversational AI"
 OR chatbot OR chatbots OR "chat bot"
 OR "dialogue system" OR "dialogue systems"
 OR "virtual agent" OR "virtual agents"
 OR "virtual coach" OR "virtual assistant" OR "virtual nurse"
 )
)
AND
(
 TI (
 cardiometabolic OR "diabetes mellitus, type 2" OR "type 2 diabetes" OR T2DM
 OR hypertension OR "high blood pressure"
 OR "heart failure"
 OR "cardiovascular disease" OR "cardiovascular diseases"
 OR "coronary heart disease" OR "ischemic heart disease"
 OR obes* OR "metabolic syndrome" OR overweight
 OR dyslipidemia OR dyslipidemias OR hyperlipidemia OR hyperlipidemias
 OR cholesterol
 )
 OR
 AB (
 cardiometabolic OR "diabetes mellitus, type 2" OR "type 2 diabetes" OR T2DM
 OR hypertension OR "high blood pressure"
 OR "heart failure"
 OR "cardiovascular disease" OR "cardiovascular diseases"
 OR "coronary heart disease" OR "ischemic heart disease"
 OR obes* OR "metabolic syndrome" OR overweight
 OR dyslipidemia OR dyslipidemias OR hyperlipidemia OR hyperlipidemias
 OR cholesterol
 )
)

Limiters applied: English Language; Published Date: 20200101–20251130.

Records retrieved before deduplication: 3.

# **IEEE Xplore**

The following search string was used in IEEE Xplore. The search was run on March 25, 2026, and results were limited to English-language journal and conference publications from 2020 onwards.

(
 ("large language model" OR "large-language model*" OR "generative AI"
 OR "generative artificial intelligence" OR "Generative Pre-trained Transformer"
 OR "foundation model" OR "foundation models"
 OR "ChatGPT" OR "GPT-4" OR "GPT-3.5"
 OR "Llama" OR "LLaMA" OR "Claude" OR "Gemini" OR "Bard" OR "deepseek"
 OR "large generative model*")
 AND
 ("conversational agent" OR "conversational agents" OR "conversational AI"
 OR chatbot OR chatbots OR "chat bot" OR "dialogue system" OR "dialogue systems"
 OR "conversational system" OR "virtual agent" OR "virtual coach" OR "virtual assistant")
 AND
 (cardiometabolic OR "type 2 diabetes" OR "diabetes mellitus type 2" OR T2DM
 OR hypertension OR "cardiovascular disease" OR "cardiovascular diseases"
 OR "coronary heart disease" OR "heart failure"
 OR obesity OR obes* OR "metabolic syndrome"
 OR overweight OR dyslipidemia OR dyslipidemias OR hyperlipidemia OR hyperlipidemias
 OR cholesterol)
)

Records retrieved before deduplication: 21.

# **ACM Digital Library**

The following search string was used in the ACM Digital Library. The search was run on March 25, 2026, and results were limited to English-language publications from 2020 to 2025.

[[All: "large language model"] OR [All: "large-language model*"]
 OR [All: "generative ai"] OR [All: "generative artificial intelligence"]
 OR [All: "generative pre-trained transformer"]
 OR [All: "foundation model"] OR [All: "foundation models"]
 OR [All: "chatgpt"] OR [All: "gpt-4"] OR [All: "gpt-3.5"]
 OR [All: "llama"] OR [All: "LLaMA"]
 OR [All: "claude"] OR [All: "gemini"] OR [All: "bard"] OR [All: "deepseek"]
 OR [All: "large generative model*"]]
AND
[[All: "conversational agent"] OR [All: "conversational agents"]
 OR [All: "conversational ai"] OR [All: chatbot] OR [All: chatbots] OR [All: "chat bot"]
 OR [All: "dialogue system"] OR [All: "dialogue systems"]
 OR [All: "conversational system"] OR [All: "virtual agent"] OR [All: "virtual coach"]
 OR [All: "virtual assistant"]]
AND
[[All: cardiometabolic] OR [All: "type 2 diabetes"] OR [All: "diabetes mellitus type 2"]
 OR [All: t2dm] OR [All: hypertension]
 OR [All: "cardiovascular disease"] OR [All: "cardiovascular diseases"]
 OR [All: "coronary heart disease"] OR [All: "ischemic heart disease"]
 OR [All: "heart failure"] OR [All: obesity] OR [All: obes*]
 OR [All: "metabolic syndrome"] OR [All: overweight]
 OR [All: dyslipidemia] OR [All: dyslipidemias]
 OR [All: hyperlipidemia] OR [All: hyperlipidemias]
 OR [All: cholesterol]]
AND
[E-Publication Date: (01/01/2020 TO 11/30/2025)]

Records retrieved before deduplication: 539.

# **arXiv**

The arXiv preprint server was searched using the native Advanced Search interface. To maximize recall within the preprint environment, a broad LLM/chatbot concept block was combined with a cardiometabolic condition block using Boolean operators in the All fields setting. No subject restriction was applied, and cross-listed papers were included. The search was limited by original submission date from January 1, 2020 to November 30, 2025 and was run on March 25, 2026.

The search was entered in the arXiv Advanced Search interface as follows:

Line 1 (All fields): ("large language model" OR "large language models" OR "large-language model" OR "large-language models" OR "generative AI" OR "generative artificial intelligence" OR "Generative Pre-trained Transformer" OR "foundation model" OR "foundation models" OR ChatGPT OR "GPT-4" OR "GPT-3.5" OR "GPT-3" OR gpt4 OR gpt3 OR gpt3.5 OR LLaMA OR "Llama 2" OR Claude OR Gemini OR Bard OR deepseek OR chatbot OR chatbots OR "chat bot")

Line 2 (AND + All fields): (cardiometabolic OR "diabetes mellitus, type 2" OR "type 2 diabetes" OR T2DM OR hypertension OR "high blood pressure" OR "heart failure" OR "cardiovascular disease" OR "cardiovascular diseases" OR "coronary heart disease" OR "ischemic heart disease" OR obes* OR "metabolic syndrome" OR overweight OR dyslipidemia OR dyslipidemias OR hyperlipidemia OR hyperlipidemias OR cholesterol)

Additional settings: Include cross-listed papers = yes; Date range = 2020-01-01 to 2025-11-30; Date type = original submission date; Subject filter = none applied.

Records retrieved before deduplication: 87.

# **ClinicalTrials.gov and WHO ICTRP**

Clinical trial registries (ClinicalTrials.gov and WHO ICTRP) were searched on March 25, 2026 to identify ongoing or recently completed studies involving large language model–driven conversational agents for cardiometabolic care. On ClinicalTrials.gov, the following settings were applied:

- Condition or disease: “type 2 diabetes” OR “hypertension” OR “cardiovascular disease” OR “heart failure” OR “obesity” OR “overweight” OR “metabolic syndrome” OR “dyslipidemia” OR “hyperlipidemia”.
- Other terms: “chatbot” OR “conversational agent” OR “conversational AI” OR “ChatGPT” OR “large language model” OR “generative AI”.
- Study type: interventional studies.
- Study start date: from January 1, 2020.

The WHO ICTRP portal was searched on the same date using the same 2 concept blocks: cardiometabolic conditions (“type 2 diabetes” OR “hypertension” OR “cardiovascular disease” OR “heart failure” OR “obesity” OR “overweight” OR “metabolic syndrome” OR “dyslipidemia” OR “hyperlipidemia”) and conversational or LLM terms (“chatbot” OR “conversational agent” OR “conversational AI” OR “ChatGPT” OR “large language model” OR “generative AI”).

Registry records from ClinicalTrials.gov and WHO ICTRP were pooled before deduplication; therefore the registry yield is reported here as a combined pre-deduplication count.

Records retrieved before deduplication from registries: 35.

# **Summary of records retrieved before deduplication**

- PubMed (MEDLINE): 65
- Web of Science Core Collection: 29
- Embase: 189
- CINAHL: 14
- APA PsycInfo: 3
- IEEE Xplore: 21
- ACM Digital Library: 539
- arXiv: 87
- Clinical trial registries: 35

Total records retrieved before deduplication: 982.
