# Supplementary material for: Behavior Change Content and Implementation of Large Language Model–Driven Conversational Agents in Cardiometabolic Care: Scoping Review
Source: J Med Internet Res. 2026 Jul 15;28:e89190. doi: 10.2196/89190 (PMC13372078; doi:10.2196/89190)
Supplement: Multimedia Appendix 2 [file jmir-v28-e89190-s002.docx]

**Multimedia Appendix 2: Mixed Methods Appraisal Tool (MMAT) assessment of empirical human-participant or evaluator-based studies**

**Supplementary Table S1. Methodological quality of included studies assessed using the Mixed Methods Appraisal Tool (MMAT, 2018 version).**

Abbreviations: CT = Cannot tell; N/A = Not applicable (study design did not involve empirical data collection from human participants, intended users, clinicians, experts, crowdworkers, or evaluators eligible for MMAT appraisal, or focused solely on technical system performance, framework development, proof-of-concept testing, or simulated/model output evaluation); RCT = Randomised controlled trial.

*MMAT Criteria (C1–C5) by Category:

Cat 1 (Randomised): C1 Randomisation approp.? C2 Groups comparable? C3 Complete outcome data? C4 Outcome assessors blinded? C5 Adherence to intervention?

Cat 2 (Non-randomised): C1 Participants representative? C2 Measurements approp.? C3 Complete outcome data? C4 Confounders accounted for? C5 Intervention administered as intended?

Cat 3 (Descriptive): C1 Sampling strategy relevant? C2 Sample representative? C3 Measurements approp.? C4 Risk of non-response bias low? C5 Statistical analysis approp.?

Cat 4 (Qualitative): C1 Is the qualitative approach appropriate to answer the research question? C2 Are the qualitative data collection methods adequate to address the research question? C3 Are the findings adequately derived from the data? C4 Is the interpretation of results sufficiently substantiated by the data? C5 Is there coherence between qualitative data sources, collection, analysis and interpretation?

Cat 5 (Mixed Methods): C1 Rationale for mixing? C2 Components effectively integrated? C3 Outputs interpreted adequately? C4 Divergences addressed? C5 Quality of quant/qual components?

| **Study (first author, year)** | **MMAT Category*** | **C1** | **C2** | **C3** | **C4** | **C5** | Study design or rationale for N/A |
| --- | --- | --- | --- | --- | --- | --- | --- |
| Abbasian et al., 2024[34] | N/A | — | — | — | — | — | Technical simulation of nutrient risk assessment agent. |
| Aguzzi et al., 2025[35] | N/A | — | — | — | — | — | Model performance benchmarking on QA datasets. |
| Ahmadi et al., 2025[36] | 5. Mixed methods | Yes | Yes | Yes | Yes | Yes | Usability study integrating quantitative scales and qualitative feedback. |
| Andreadis et al., 2024[37] | 5. Mixed methods | Yes | Yes | Yes | Yes | Yes | Participatory design workshops and survey evaluation. |
| Antia et al., 2025[38] | 2. Quantitative non-randomised | Yes | CT | Yes | Yes | Yes | Single-arm pilot trial (pre–post); confounding (C2) difficult to assess without control. |
| Cheng et al., 2025[39] | 1. Quantitative randomised | Yes | Yes | Yes | Yes | Yes | Four-arm randomised factorial experiment on chatbot phrasing. |
| Chuang et al., 2025[40] | N/A | — | — | — | — | — | System accuracy testing on benchmark cases. |
| Coleman et al., 2025[41] | 1. Quantitative randomised | Yes | Yes | Yes | Yes | Yes | Non-inferiority feasibility RCT of digital clinician vs nurse. |
| Dao et al., 2024[42] | N/A | — | — | — | — | — | System evaluation on synthetic profiles and test questions. |
| Đurković et al., 2025[43] | N/A | — | — | — | — | — | Technical proof-of-concept ECG/ChatGPT system evaluation; volunteer testing was described, but volunteer count and participant-level outcomes were not reported. |
| Elfayoumi et al., 2025[44] | N/A | — | — | — | — | — | Prediction model accuracy evaluation. |
| Gollapalli et al., 2025[45] | 3. Quantitative descriptive | Yes | CT | Yes | Yes | Yes | Crowdsourced ratings; representativeness of crowdworkers (C2) for patient population unclear. |
| Huang et al., 2025[46] | 3. Quantitative descriptive | Yes | CT | Yes | Yes | Yes | Online rating experiment; sampling strategy (C2) unclear regarding target population representativeness. |
| Hussain et al., 2025[47] | N/A | — | — | — | — | — | Quality evaluation of LLM answers to static questions. |
| Jeon et al., 2025[48] | 5. Mixed methods | Yes | Yes | Yes | Yes | Yes | Formative evaluation using surveys, log analysis and interviews. |
| Kelly et al., 2025[49] | N/A | — | — | — | — | — | Technical evaluation of RAG system; simulated queries only. |
| Kozaily et al., 2023[50] | N/A | — | — | — | — | — | Technical evaluation of ChatGPT-3.5 and Bard answers to 30 simulated heart failure patient questions. |
| Liang et al., 2025[51] | 1. Quantitative randomised | Yes | Yes | Yes | Yes | Yes | Online randomised experiment comparing customisable vs baseline agent. |
| Meng et al., 2025 (Eval)[52] | 5. Mixed methods | Yes | Yes | Yes | Yes | Yes | Quantitative physician ratings integrated with qualitative interviews. |
| Meng et al., 2025 (T2MD)[53] | 5. Mixed methods | Yes | Yes | Yes | Yes | Yes | Convergent design combining controlled app evaluation with interviews. |
| Mohd Dan et al., 2025[54] | 1. Quantitative randomised | Yes | Yes | Yes | Yes | Yes | Two-arm randomised controlled trial comparing NExGEN personalised prompt generation plus ChatGPT vs structured manual ChatGPT guidance for weight management. |
| Montagna et al., 2023[55] | N/A | — | — | — | — | — | System architecture description and illustrative case study. |
| Mustafa et al., 2025[56] | 3. Quantitative descriptive | CT | CT | Yes | Yes | Yes | Cross-sectional moderated question-and-answer study of 51 adults with diabetes; convenience clinical sampling limits representativeness. |
| Neary et al., 2025[57] | N/A | — | — | — | — | — | Methods/framework-development study; initial framework testing involved 5 evaluators rating 12 dialogues, but no patient-participant sample size or participant-level outcomes were reported. |
| Pan, 2025[58] | 4. Qualitative | Yes | Yes | Yes | Yes | Yes | Autoethnographic self-study; rigorous data collection and interpretation. |
| Patil et al., 2025[59] | N/A | — | — | — | — | — | System latency and response relevance technical testing. |
| Pay et al., 2025[60] | N/A | — | — | — | — | — | Technical evaluation of ChatGPT-4o, Gemini, and Bing responses to 50 frequently asked CAD questions. |
| Ponzo et al., 2024[61] | N/A | — | — | — | — | — | Comparative technical evaluation of 10 general-purpose AI chatbots across 2 hypothetical nutrition cases. |
| Rodriguez et al., 2024[62] | N/A | — | — | — | — | — | Prototype development and scenario-based provider review. |
| Rossi et al., 2024[63] | N/A | — | — | — | — | — | Diagnostic classifier evaluation on retrospective dataset. |
| Saraç et al., 2025[64] | 3. Quantitative descriptive | Yes | CT | Yes | Yes | Yes | Expert trainer ratings of generated programmes; sampling of experts (C2) not detailed. |
| Strömel et al., 2024[65] | 5. Mixed methods | Yes | Yes | Yes | Yes | Yes | Online experiment with interview pre-study evaluating LLM-generated fitness-tracker narratives. |
| Szymanski et al., 2024[66] | 5. Mixed methods | Yes | Yes | Yes | Yes | Yes | Mixed-methods dietitian validation and prototype-refinement study; 12 RDs participated in initial validation and focus-group participants were reported separately. |
| Tayal et al., 2025 (Food)[67] | 2. Quantitative non-randomised | Yes | Yes | Yes | Yes | Yes | Within-subject crossover comparison of two systems. |
| Tayal et al., 2025 (HF)[68] | N/A | — | — | — | — | — | Evaluation of synthetic dialogues generated by ChatGPT. |
| Vats et al., 2025[69] | N/A | — | — | — | — | — | ML model performance metrics (accuracy, F1) only. |
| Wali et al., 2024[70] | N/A | — | — | — | — | — | Recommender system development and demonstration. |
| Wang et al., 2025[71] | 2. Quantitative non-randomised | Yes | Yes | Yes | Yes | Yes | Two-phase benchmarking and prospective external-validation study comparing Cascade Agent with baseline models and physicians. |
