# Supplementary material for: Behavior Change Content and Implementation of Large Language Model–Driven Conversational Agents in Cardiometabolic Care: Scoping Review
Source: J Med Internet Res. 2026 Jul 15;28:e89190. doi: 10.2196/89190 (PMC13372078; doi:10.2196/89190)
Supplement: Multimedia Appendix 3 [file jmir-v28-e89190-s003.docx]

**Multimedia Appendix 3: Detailed mapping of confirmed behaviour change techniques and delivery-level classifications**

**Supplementary Table S2. Confirmed behaviour change technique (BCT) coding and delivery-level classification across 38 included studies**

*Note.* This submission-oriented appendix table retains only confirmed BCTs with sufficiently clear textual support. Candidate or borderline codes were retained in the accompanying Excel working sheet but were not carried into this concise appendix table. Delivery levels are reported as Level 0 (static/non-adaptive), Level 1 (rule-based/scripted/structured digital support), Level 2 (generative or dynamically tailored support), or Mixed mode when the same BCT appeared through more than one delivery mechanism within the same study. Where relevant, the component levels underlying a Mixed mode classification are indicated in parentheses. BCT labels were normalised to BCTTv1 terminology for consistency across studies.

| **Study** | Evidence/source type for BCT coding | **Confirmed BCT(s)** | **Delivery level(s)** | **Brief evidence note** |
| --- | --- | --- | --- | --- |
| Abbasian et al., 2024[34] (openCHA) | System or technical evaluation | 2.3 Self-monitoring of behaviour; 2.2 Feedback on behaviour; 4.1 Instruction on how to perform behaviour; 5.1 Information about health consequences; 9.1 Credible source | 2.3: Level 1; 2.2: Level 2; 4.1: Level 2; 5.1: Level 2; 9.1: Level 2 | 2.3: Users input meal/intake information for assessment. 2.2: Agent analyzes intake and flags nutritional risk/problem areas. 4.1: Provides dietary self-management guidance. 5.1: Explains why certain nutritional patterns are problematic for diabetes management. 9.1: Grounded in ADA dietary guidance and nutrition databases. |
| Aguzzi et al., 2025[35] | Technical evaluation | 4.1 Instruction on how to perform behaviour; 5.1 Information about health consequences | 4.1: Level 2; 5.1: Level 2 | 4.1: Answers patient questions about hypertension management and what to do. 5.1: Explains disease- and treatment-relevant consequences for lay users. |
| Ahmadi et al., 2025[36] (EFTeacher) | Intervention development or pilot | 15.2 Mental rehearsal of successful performance; 8.1 Behavioural practice/rehearsal; 2.2 Feedback on behaviour | 15.2: Mixed mode (Levels 1+2); 8.1: Level 1; 2.2: Level 2 | 15.2: Core task is vivid future-event cue generation to support healthier choices. 8.1: Users repeatedly practice generating/refining EFT cues. 2.2: Chatbot iteratively guides revision of user-generated cues. |
| Andreadis et al., 2024[37] | Early development or design study | 7.1 Prompts/cues | 7.1: Level 2 | 7.1: Automated patient follow-up messages are a stated functionality. |
| Antia et al., 2025[38] (Healthy Heart Assistant) | Single-arm empirical human study | 7.1 Prompts/cues; 4.1 Instruction on how to perform behaviour; 5.1 Information about health consequences | 7.1: Level 1; 4.1: Level 1; 5.1: Level 1 | 7.1: Medication and appointment reminders were explicitly set up. 4.1: Education on hypertension self-care and lifestyle modification. 5.1: Knowledge testing and education covered risks/symptoms/management consequences. |
| Cheng et al., 2025[39] | Empirical human study or HCI experiment | 4.1 Instruction on how to perform behaviour; 3.3 Social support (emotional) | 4.1: Mixed mode (Levels 0+1); 3.3: Level 1 | 4.1: Dialogue content includes weight-management recommendations. 3.3: Caring talk is explicitly introduced to convey concern and care. |
| Chuang et al., 2025[40] | System or technical study | 2.3 Self-monitoring of behaviour; 2.2 Feedback on behaviour; 4.1 Instruction on how to perform behaviour; 5.1 Information about health consequences; 9.1 Credible source | 2.3: Level 1; 2.2: Level 2; 4.1: Level 2; 5.1: Level 2; 9.1: Level 2 | 2.3: Users submit diet, medication, and health-related input data. 2.2: System returns personalized analysis of diet/medication/health reports. 4.1: Provides personalized health and lifestyle advice. 5.1: Explains links between lifestyle, medication, and chronic disease risks. 9.1: Uses RAG with authoritative chronic disease knowledge sources. |
| Coleman et al., 2025[41] | Feasibility RCT | 4.1 Instruction on how to perform behaviour; 2.2 Feedback on behaviour; 5.1 Information about health consequences | 4.1: Level 1; 2.2: Level 1; 5.1: Level 1 | 4.1: Tutorial covers pen preparation, injection, dosing, storage, and side effects. 2.2: Interactive checking of understanding and corrections during tutorial. 5.1: Education includes side effects and medication implications. |
| Dao et al., 2024[42] | System design or workshop paper | 4.1 Instruction on how to perform behaviour; 7.1 Prompts/cues; 3.3 Social support (emotional); 2.2 Feedback on behaviour | 4.1: Level 2; 7.1: Level 2; 3.3: Level 2; 2.2: Level 2 | 4.1: AI chatbot gives tailored advice on prevention behaviours. 7.1: Reminder and activity system issues personalized reminders/prompts. 3.3: Emotional support module provides motivational/emotional support content. 2.2: Tailored guidance responds to user context and tracking input. |
| Đurković et al., 2025[43] (CardiaTalker) | Technical/proof-of-concept system evaluation | 2.6 Biofeedback; 5.1 Information about health consequences; 9.1 Credible source | 2.6: Level 2; 5.1: Level 2; 9.1: Mixed mode | 2.6: ECG biosignals are translated into conversational AI interpretation for users. 5.1: System provides general educational insights into cardiac health and rhythm patterns. 9.1: System can provide both clinician opinion and AI interpretation in parallel. |
| Elfayoumi et al., 2025[44] | Technical evaluation | 2.7 Feedback on outcome(s) of behaviour; 5.1 Information about health consequences; 9.1 Credible source | 2.7: Level 2; 5.1: Level 2; 9.1: Level 2 | 2.7: Outputs individualized diabetes likelihood and confidence based on patient indicators. 5.1: Explains why indicators such as HbA1c, glucose, BMI, and smoking matter. 9.1: RAG-enhanced with guidelines and similar cases to support explanation. |
| Gollapalli et al., 2025[45] (PIRsuader) | Technical/dialogue generation study | 5.1 Information about health consequences; 9.1 Credible source; 11.2 Reduce negative emotions; 3.3 Social support (emotional); 1.2 Problem solving | 5.1: Level 2; 9.1: Level 2; 11.2: Level 2; 3.3: Level 2; 1.2: Level 2 | 5.1: Dialog acts include logical appeal and insulin information about complications and benefits. 9.1: Credibility appeal explicitly uses expert endorsement or medical authority. 11.2: Core purpose is to reduce fear and concern about insulin initiation. 3.3: Designed for empathetic, persuasive counselling conversations. 1.2: Suggests solutions/reasons to overcome barriers to insulin use. |
| Huang et al., 2025[46] | Empirical human study | 3.3 Social support (emotional); 4.1 Instruction on how to perform behaviour | 3.3: Level 2; 4.1: Level 2 | 3.3: Participants noted empathy/supportive tone as a valued feature of AI messages. 4.1: Coaching messages provide concrete weight-control advice and suggestions. |
| Hussain et al., 2025[47] | Evaluation study | 4.1 Instruction on how to perform behaviour; 5.1 Information about health consequences | 4.1: Level 2; 5.1: Level 2 | 4.1: Queries and answers cover diet, exercise, insulin use, and self-management actions. 5.1: Evaluates explanations around glycemic interpretation and diabetes risks. |
| Jeon et al., 2025[48] (DTalksBot) | Formative qualitative human study | 4.1 Instruction on how to perform behaviour; 5.1 Information about health consequences; 3.3 Social support (emotional); 2.2 Feedback on behaviour; 9.1 Credible source | 4.1: Level 2; 5.1: Level 2; 3.3: Level 2; 2.2: Level 2; 9.1: Level 2 | 4.1: Patients asked about diet, exercise, medication timing, and daily management. 5.1: High volume of questions on complications/comorbidities and symptom meaning. 3.3: Mental health management/support emerged as a distinct theme; empathy/support assessed. 2.2: Users input individualized blood glucose and treatment context for tailored responses. 9.1: RAG grounded in curated, verified diabetes knowledge sources. |
| Kelly et al., 2025[49] | Development and evaluation study | 4.1 Instruction on how to perform behaviour; 5.1 Information about health consequences; 9.1 Credible source | 4.1: Level 2; 5.1: Level 2; 9.1: Level 2 | 4.1: Responds to T2DM patient queries to support education and self-management. 5.1: Explains disease implications and complications in health-literacy terms. 9.1: Explicit source attribution to validated reference documents / general knowledge flagging. |
| Kozaily et al., 2023[50] | Evaluation study | 4.1 Instruction on how to perform behaviour; 5.1 Information about health consequences | 4.1: Level 2; 5.1: Level 2 | 4.1: Responses addressed management, monitoring, and lifestyle practices in HF. 5.1: Questions/responses covered prognosis, symptoms, and implications of HF management. |
| Liang et al., 2025[51] (SmartEats) | System or recommendation study | 4.1 Instruction on how to perform behaviour; 5.1 Information about health consequences; 2.2 Feedback on behaviour | 4.1: Level 2; 5.1: Level 2; 2.2: Level 2 | 4.1: Provides concrete food recommendations and how to adjust meals. 5.1: Explains why options fit health goals and likely health implications. 2.2: Uses user context/goals to give tailored explanatory feedback on choices. |
| Meng et al., 2025 (Eval)[52] (Between Knowledge and Care) | Evaluation study | 4.1 Instruction on how to perform behaviour; 5.1 Information about health consequences | 4.1: Level 2; 5.1: Level 2 | 4.1: Messages and responses include diet and activity advice. 5.1: Responses explain risks, HbA1c meaning, and self-care consequences. |
| Meng et al., 2025 (T2MD)[53] (T2MD Health) | Empirical human study or app pilot | 2.3 Self-monitoring of behaviour; 2.4 Self-monitoring of outcomes of behaviour; 2.2 Feedback on behaviour; 7.1 Prompts/cues; 4.1 Instruction on how to perform behaviour; 5.1 Information about health consequences; 3.2 Social support (practical) | 2.3: Level 1; 2.4: Level 1; 2.2: Level 1; 7.1: Level 1; 4.1: Mixed mode (Levels 1+2); 5.1: Level 2; 3.2: Level 1 | 2.3: Daily health tracking and interaction logs. 2.4: Monthly reports include glucose trends, symptoms, and adherence-related summaries. 2.2: Monthly reports and AI summaries highlight knowledge gaps and behaviour patterns. 7.1: Reminder functions are explicitly described. 4.1: Structured educational content plus GPT-4o personalized explanations/advice. 5.1: AI explains implications of regimens and disease-management decisions. 3.2: Escalation/notification and support workflow may provide practical support. |
| Mohd Dan et al., 2025[54] | Randomized controlled trial | 1.1 Goal setting (behaviour); 1.2 Problem solving; 1.3 Goal setting (outcome); 1.4 Action planning; 2.2 Feedback on behaviour; 2.3 Self-monitoring of behaviour; 4.1 Instruction on how to perform behaviour; 9.1 Credible source | 1.1: Level 2; 1.2: Level 2; 1.3: Level 2; 1.4: Level 2; 2.2: Level 2; 2.3: Level 1; 4.1: Mixed mode (Levels 0+1+2); 9.1: Level 2 | 1.1: Participants established individualized weight-loss related behavioural targets. 1.2: Ongoing dialogue addressed barriers, challenges, and strategy refinements. 1.3: Individualized weight-loss targets were explicitly set. 1.4: Structured weekly objectives and specific behavioural tasks were assigned. 2.2: Dynamic prompt-response cycles produced personalized behavioural feedback. 2.3: Adherence and progress involved daily data logging/self-tracking. 4.1: Intervention included dynamic personalized outputs (L2), structured manual prompts (L1), and static non-adaptive content (L0). 9.1: Health-specialist–framed intervention logic and clinically grounded recommendations. |
| Montagna et al., 2023[55] | Architecture or prototype | 7.1 Prompts/cues; 2.3 Self-monitoring of behaviour; 2.4 Self-monitoring of outcomes of behaviour; 2.2 Feedback on behaviour; 4.1 Instruction on how to perform behaviour | 7.1: Level 1; 2.3: Level 1; 2.4: Level 1; 2.2: Level 1; 4.1: Level 1 | 7.1: Regular reminders are explicitly described. 2.3: Prototype motivates patients to acquire BP measures and follow prescriptions. 2.4: Prototype supports monitoring the evolution of blood pressure over time. 2.2: Motivational messages are based on adherence/performance patterns. 4.1: Chatbot is intended to support day-to-day self-management actions. |
| Mustafa et al., 2025[56] | Empirical human study | 3.3 Social support (emotional); 4.1 Instruction on how to perform behaviour; 5.1 Information about health consequences | 3.3: Level 2; 4.1: Level 2; 5.1: Level 2 | 3.3: Participants perceived answers as empathetic/supportive. 4.1: Answers covered blood sugar management, medication use, diet, and lifestyle management. 5.1: Questions/answers included complications and health implications of diabetes management. |
| Neary et al., 2025[57] (Coach Iris FAST) | Methods or evaluation framework paper | 4.1 Instruction on how to perform behaviour | 4.1: Level 2 | 4.1: Coach Iris can advise on healthy lifestyle strategies, behaviour change, nutrition, exercise, and wellbeing. |
| Pan, 2025[58] | Autoethnography | 2.6 Biofeedback; 2.3 Self-monitoring of behaviour; 2.4 Self-monitoring of outcomes of behaviour; 2.2 Feedback on behaviour; 4.1 Instruction on how to perform behaviour; 5.1 Information about health consequences | 2.6: Level 2; 2.3: Level 1; 2.4: Level 1; 2.2: Level 2; 4.1: Level 2; 5.1: Level 2 | 2.6: CGM is used as a first-class signal and discussed through chatbot interpretation. 2.3: Meals, exercise, caffeine, alcohol, naps, and work events are tracked. 2.4: CGM, HR/HRV, sleep, BP, weight, and anxiety are monitored. 2.2: Chatbot provides personalized reflections after salient events. 4.1: Chatbot offers personalized lifestyle recommendations. 5.1: System explains abnormal glucose fluctuations in relation to behaviours. |
| Patil et al., 2025[59] (MedBot) | Technical study | 4.1 Instruction on how to perform behaviour; 5.1 Information about health consequences; 9.1 Credible source | 4.1: Level 2; 5.1: Level 2; 9.1: Level 2 | 4.1: Provides patient-specific guidance and treatment suggestions for heart disease management. 5.1: Responds to disease-, symptom-, and treatment-related inquiries. 9.1: Knowledge-base interaction module connects to medical repositories and medicine databases. |
| Pay et al., 2025[60] | Evaluation study | 4.1 Instruction on how to perform behaviour; 5.1 Information about health consequences; 9.1 Credible source | 4.1: Level 2; 5.1: Level 2; 9.1: Level 2 | 4.1: Questions included what to do, treatment, self-care, ED attendance, and dietary advice. 5.1: Responses concerned symptoms, risk factors, prevention, recovery, and complications. 9.1: Question set and evaluation referenced AHA/NHLBI-aligned cardiovascular information sources. |
| Ponzo et al., 2024[61] | Evaluation study | 4.1 Instruction on how to perform behaviour; 5.1 Information about health consequences | 4.1: Level 2; 5.1: Level 2 | 4.1: Chatbots were asked to generate tailored dietary plans and advice. 5.1: Evaluation included caloric targets, macronutrient adequacy, and healthfulness of recommendations. |
| Rodriguez et al., 2024[62] | Early development or design study | 7.1 Prompts/cues; 2.7 Feedback on outcome(s) of behaviour; 4.1 Instruction on how to perform behaviour | 7.1: Level 2; 2.7: Level 2; 4.1: Level 2 | 7.1: 24/7 SMS platform promotes engagement and patient interaction. 2.7: Responds to reported RPM values and generates follow-up conversations based on monitored results. 4.1: Patient-facing educational content is a key functional requirement. |
| Rossi et al., 2024[63] (DiabeTalk) | Technical comparative study | 2.7 Feedback on outcome(s) of behaviour; 5.1 Information about health consequences | 2.7: Level 2; 5.1: Level 2 | 2.7: Chatbot predicts diabetes type from symptoms/history and reports result conversationally. 5.1: Explains links between symptoms, lifestyle factors, and diabetes type. |
| Saraç et al., 2025[64] | Comparative evaluation study | 4.1 Instruction on how to perform behaviour; 1.4 Action planning | 4.1: Level 2; 1.4: Level 2 | 4.1: Chatbots generate personalized exercise programs. 1.4: Exercise programs specify frequency/intensity/time/type planning elements. |
| Strömel et al., 2024[65] | Empirical HCI study | 2.2 Feedback on behaviour; 13.2 Framing/reframing | 2.2: Level 2; 13.2: Level 2 | 2.2: LLM transforms step data into qualitative descriptions that help users interpret behaviour patterns. 13.2: Narrative representation changes how users perceive numeric tracker data and supports reflection. |
| Szymanski et al., 2024[66] | Mixed-methods dietitian validation and prototype-refinement study | 4.1 Instruction on how to perform behaviour; 5.1 Information about health consequences; 9.1 Credible source | 4.1: Level 2; 5.1: Level 2; 9.1: Level 2 | 4.1: Prototype explains whether a product is suitable and what to choose/do instead. 5.1: Outputs describe healthful/less healthful aspects of products relative to goals. 9.1: Uses dietitian-reviewed template instructions and authoritative nutrition content (labels/guidelines). |
| Tayal et al., 2025 (Food)[67] | Within-subject patient comparison study | 4.1 Instruction on how to perform behaviour; 5.1 Information about health consequences; 2.2 Feedback on behaviour; 9.1 Credible source | 4.1: Mixed mode (Levels 1+2); 5.1: Mixed mode (Levels 1+2); 2.2: Mixed mode (Levels 1+2); 9.1: Level 1 | 4.1: Both systems tell patients how to interpret/manage foods in relation to sodium; NS is more rule/database-driven, GPT is generative. 5.1: Responses connect sodium content to HF self-care and risk implications. 2.2: Systems respond to the patient’s own queried foods and portion context. 9.1: Neurosymbolic system is grounded in USFDC food composition data. |
| Tayal et al., 2025 (HF)[68] | Feasibility or synthetic dataset study | 4.1 Instruction on how to perform behaviour; 5.1 Information about health consequences | 4.1: Level 2; 5.1: Level 2 | 4.1: Generated dialogues target food, exercise, and fluid intake self-care strategies. 5.1: Conversations aim to explain why self-care actions matter in heart failure. |
| Vats et al., 2025[69] | Technical study | 4.1 Instruction on how to perform behaviour; 5.1 Information about health consequences | 4.1: Level 2; 5.1: Level 2 | 4.1: Provides lifestyle modification guidance around diet, exercise, sleep, and symptom-related support. 5.1: Links 13 clinical factors with CAD risk and management relevance. |
| Wali et al., 2024[70] | Technical study | 2.7 Feedback on outcome(s) of behaviour; 5.1 Information about health consequences; 7.1 Prompts/cues | 2.7: Mixed mode (Levels 1+2); 5.1: Level 2; 7.1: Level 1 | 2.7: Generates patient-specific risk reports/bar charts and chatbot explanations based on classifier output. 5.1: Explains reasons behind high/low risk and relevant heart-attack factors. 7.1: Warning notifications are generated when risk factors are out of range. |
| Wang et al., 2025[71] (Cascade) | Two-phase benchmarking and external-validation study | 4.1 Instruction on how to perform behaviour; 5.1 Information about health consequences; 2.6 Biofeedback; 3.3 Social support (emotional); 9.1 Credible source | 4.1: Level 2; 5.1: Level 2; 2.6: Level 2; 3.3: Level 2; 9.1: Level 2 | 4.1: Structured hypertension education workflow provides prevention, lifestyle, and management guidance. 5.1: Knowledge base covers pathophysiology, risk factors, prevention, medication adherence, and outcomes. 2.6: Framework interprets BP classification and risk stratification from patient data. 3.3: External validation explicitly assessed and improved emotional support. 9.1: Grounded in Chinese hypertension guidelines, authoritative texts, and specialist-verified Q&A. |
