# Supplementary material for: Behavior Change Content and Implementation of Large Language Model–Driven Conversational Agents in Cardiometabolic Care: Scoping Review
Source: J Med Internet Res. 2026 Jul 15;28:e89190. doi: 10.2196/89190 (PMC13372078; doi:10.2196/89190)
Supplement: Multimedia Appendix 4 [file jmir-v28-e89190-s004.docx]

**Multimedia Appendix 4: Detailed characteristics of interventions, comparators, duration, and reported outcomes**

**Supplementary Table S3. Intervention, comparator, duration and outcomes for included studies**

Abbreviations: RAG, retrieval-augmented generation; LLM, large language model; ECG, electrocardiogram; EHR, electronic health record; ML, machine learning; AUC, area under the receiver operating characteristic curve; BMI, body mass index; SDOH, social determinants of health; ACSM, American College of Sports Medicine; NASM, National Academy of Sports Medicine.

| **Study (first author, year)** | **Journal or conference** | **Intervention or system** | **Comparator** | **Duration or follow-up** | **Outcomes measured** |
| --- | --- | --- | --- | --- | --- |
| Abbasian et al., 2024[34] | IEEE EMBC 2024 | Knowledge-infused conversational health agent using GPT-3.5-turbo with Nutritionix retrieval and diabetes guidelines | GPT-4 baseline configuration | Single-turn question-and-answer interactions | Nutrient risk assessment accuracy across seven nutrients |
| Aguzzi et al., 2025[35] | Journal of Medical Systems | Privacy-preserving hypertension self-management chatbot based on open small language models with RAG | Small language model prompts without retrieval; alternative retrieval strategies | Single-session offline evaluation | Automatic faithfulness and medical faithfulness metrics; physician ratings of answer quality |
| Ahmadi et al., 2025[36] | arXiv preprint | EFTeacher AI-powered episodic future thinking chatbot for obesity-related behaviour change | None (single-arm) | Single-session AI-assisted interview covering seven future time frames | System Usability Scale scores; ratings of episodic future thinking cue characteristics (liking, importance, excitement, vividness); qualitative user experience themes |
| Andreadis et al., 2024[37] | Studies in Health Technology and Informatics | Generative AI remote patient monitoring assistant for hypertension (patient-facing chatbot, clinician-facing smart summaries) | None | One-time online survey-based evaluation | Ratings of empathy, clarity, informativeness and usefulness of AI-generated messages and summaries; qualitative feedback on tone, structure and desired features |
| Antia et al., 2025[38] | Mayo Clinic Proceedings: Digital Health | Healthy Heart Assistant (WhatsApp-based GPT chatbot for hypertension self-care) | None (single-arm pilot) | 30-day follow-up | Feasibility, usability and acceptability; hypertension knowledge; medication adherence; office blood pressure |
| Cheng et al., 2025[39] | Proc ACM Hum-Comput Interact (CSCW) | Weight-management advice chatbot using ManyChat and GPT API with different phrase styles and caring-talk options | Four experimental conditions (explicit/implicit phrasing × caring talk/no caring talk) | Single remote session plus optional interview | Interaction satisfaction and enjoyment; misinterpretation and perceived purpose; intention to follow recommendations; interaction duration; qualitative themes on comfort, perceived care and willingness to engage |
| Chuang et al., 2025[40] | Mobile Networks and Applications | Generative-AI health advisory system delivered via LINE chatbot using ChatGPT API and retrieval | None | System-level inference evaluation (no real-time follow-up) | System accuracy for dietary, medication and health report modules; latency; classification of error types |
| Coleman et al., 2025[41] | JMIR Diabetes | “Digital Clinician” avatar-based conversational education system for semaglutide self-injection | Human nurse-led education | Single 10–15 minute educational session with post-test and two-week follow-up | Knowledge about semaglutide; self-efficacy; satisfaction; trust; usability; qualitative feedback on experience |
| Dao et al., 2024[42] | ACM AIQAM 2024 (ICMR workshop) | LLM-based diabetes prevention chatbot using GPT-3.5 with Assistants API and optional retrieval | Alternative configurations (fine-tuned only; fine-tuned plus retrieval; Assistants API-based designs) | System-level evaluation on test questions (no human participants) | Response relevance; personalisation; context-awareness; reliability across prompting conditions |
| Đurković et al., 2025[43] | MECO 2025 (Mediterranean Conference on Embedded Computing) | CardiaTalker proof-of-concept system combining IoT ECG wearable hardware with a ChatGPT-based interpretation and educational feedback pipeline | Cardiologist ECG interpretation | Prototype operational testing with volunteer ECG recordings; volunteer count not reported | Coherence of LLM-generated interpretations; latency; system reliability; feasibility of patient-facing conversational ECG feedback; no participant-level outcomes reported |
| Elfayoumi et al., 2025[44] | IEEE IMCOM 2025 | Knowledge-augmented diabetes prediction chatbot combining LLM and retrieval-based decision support | Baseline language models without retrieval or advanced prompting | Single-turn prediction and explanation | Predictive accuracy, precision, recall and F1-score; quality of explanatory output |
| Gollapalli et al., 2025[45] | COLING 2025 (International Conference on Computational Linguistics) | PIRsuader persuasive counselling chatbot for insulin initiation | Alternative dialog-act policies based on GPT-4o-mini and reinforcement learning | Single simulated counselling session per dialogue | Crowd-rated willingness to try insulin; perceived persuasiveness; empathy; specificity; question answering; persistence |
| Huang et al., 2025[46] | Journal of Technology in Behavioral Science | ChatGPT-generated behavioural weight-loss coaching messages based on participant data scenarios | Human coach-generated messages for the same scenarios | Single rating session (10 messages per phase) | Perceived helpfulness; ability to identify AI versus human messages; qualitative feedback on tone and content |
| Hussain et al., 2025[47] | arXiv preprint | ChatGPT-3.5 and GPT-4 answers to diabetes self-management questions across diet, exercise, glycaemic events and insulin handling | Comparison between ChatGPT-3.5 and GPT-4 with spot checks using other models | Single evaluation wave with offline analysis | Consistency, reliability and accuracy of advice; identification of systematic error patterns; development of a risk-tier framework |
| Jeon et al., 2025[48] | JMIR Formative Research | DTalksBot generative AI diabetes chatbot (GPT-4 with RAG over curated resources) | None (single-arm formative evaluation) | Single 60-minute patient session and single 90-minute specialist session | Types and themes of patient questions; usability; credibility; perceived social support, empathy, trust and satisfaction; clinicians’ assessment of accuracy, contextual relevance, safety and potential roles |
| Kelly et al., 2025[49] | Journal of Medical Internet Research | Retrieval-augmented generative AI chatbot for diabetes health literacy (RAG with GPT-4o mini) | None (single-arm evaluation) | Single-session evaluation (no follow-up) | Response appropriateness; source attribution accuracy; response consistency; retrieval difficulty classification |
| Kozaily et al., 2023[50] | medRxiv preprint | ChatGPT-3.5 and Bard responses to 30 commonly asked patient questions about heart failure derived from patient forums and clinician experience | ChatGPT-3.5 versus Bard | Single-time technical evaluation; each question asked 3 times between June 1 and June 24, 2023 | HF expert grading of adequacy/accuracy, consistency across repeated prompts, reviewer concordance, comparison of ChatGPT versus Bard |
| Liang et al., 2025[51] | CUI 2025 (ACM Conference on Conversational User Interfaces) | SmartEats GPT-4-powered conversational recommender system for personalised dietary advice | Baseline non-customisable conversational agent | Single 17–19 minute interaction plus one-week follow-up survey | Conversation engagement; interaction experience (enjoyment, trust, style satisfaction, intention to use); perceived recommendation quality; one-week recall and self-reported adherence; reported barriers to adherence |
| Meng et al., 2025 (Eval)[52] | arXiv preprint | LLM-generated interactive diabetes information using ChatGPT, DeepSeek-R1, Kimi K2 and ERNIE Bot 4.5 | None | Cross-sectional two-phase evaluation | Physician ratings of accuracy, safety, clarity, integrity and action orientation; qualitative interview reflections; patient usage patterns |
| Meng et al., 2025 (T2MD)[53] | arXiv preprint | T2MD Health AI-driven personalised mobile application integrating transcription, terminology explanation, conversational tracking, question-and-answer support, reminders, and summary reports | Simulated clinical visits and follow-up without access to T2MD Health | 4-week app use between baseline and follow-up plus two simulated visits | Change in diabetes knowledge; system usability; physician ratings of AI-generated reports (accuracy, relevance, readability, user-friendliness); patient self-management behaviours and experiences; technical performance indicators |
| Mohd Dan et al., 2025[54] | medRxiv preprint | NExGEN personalised prompt generator integrated with ChatGPT (o3) to deliver tailored exercise plans, 7-day diet plans, grocery lists, and meal preparation guidance | Structured manual ChatGPT prompts without personalised assessment-driven prompt generation | 12-week intervention plus 12-week maintenance/follow-up; assessments at baseline, 12 weeks, and 24 weeks | Body weight, body composition, waist circumference, dietary intake, physical activity, HbA1c, lipids, glucose, blood pressure, qualitative experience feedback |
| Montagna et al., 2023[55] | ACM GoodIT ’23 | HypertensionBot Telegram chatbot integrated with a decentralised personal data store and GPT-3 for empathic conversation | None | Continuous prototype use for home blood pressure monitoring (no predefined follow-up) | Feasibility of decentralised architecture; ability to acquire and store blood pressure measures; provision of reminders, adherence information and alerts |
| Mustafa et al., 2025[56] | medRxiv preprint | Moderated ChatGPT-based diabetes education question-and-answer support for adults with type 2 diabetes and diabetic retinopathy; interpreter support for non-English-speaking or visually impaired participants | None | Single cross-sectional session (15–45 min); 137 patient questions from 51 participants | Participant ratings of informativeness/detail/empathy; specialist ratings of clarity, completeness, correctness, safety, and recency; ICC and Fleiss kappa |
| Neary et al., 2025[57] | Frontiers in Digital Health | Coach Iris generative AI conversational health coach and FAST framework for evaluating fidelity, accuracy, safety and tone in dialogue quality | None | Framework development, initial evaluator testing with 5 evaluators rating 12 dialogues, training and ongoing monitoring procedures | FAST quality metrics; evaluator consistency processes; clinical risk escalation procedures; patient-user base size not reported |
| Pan, 2025[58] | ICHEC 2025 (International Conference on Human-Engaged Computing) | Multimodal continuous glucose monitoring and chatbot system combining wearable sensors and LLM-based conversational support | None (single-case autoethnography) | 6 weeks (42 consecutive days) | BMI; systolic blood pressure; Self-Rating Anxiety Scale scores; sleep duration; heart rate variability; continuous glucose metrics; qualitative themes on health management |
| Patil et al., 2025[59] | IEEE SENNET 2025 | MedBot conversational cardiac health assistant using the BioMistral language model | None | Single-turn or short multi-turn interactions | Response relevance; latency; accuracy of symptom interpretation |
| Pay et al., 2025[60] | Turk Kardiyol Dern Ars | ChatGPT-4o, Gemini, and Bing responses to 50 frequently asked patient questions about coronary artery disease | Cross-chatbot comparison (ChatGPT-4o vs Gemini vs Bing) | Each question asked twice, 1 week apart | Accuracy categories (comprehensive/correct, incomplete/partially correct, mixed, inaccurate/irrelevant) and reproducibility by question subgroup |
| Ponzo et al., 2024[61] | Journal of Clinical Medicine | Ten general-purpose AI chatbots generating dietary advice for 2 obesity-related cases (simple obesity; obesity with T2DM, CKD, and sarcopenia) | Cross-chatbot comparison across 10 systems | Two prompts entered on 3 consecutive days (June 27–29, 2024) | Accuracy, completeness, reproducibility, internal consistency, and nutritional adequacy of generated meal plans |
| Rodriguez et al., 2024[62] | IEEE ICHI 2024 | Generative AI-powered remote patient monitoring assistant based on GPT-4 integrated with EHR workflow | None | Scenario-based evaluation only | Provider-rated accuracy, empathy and readability of AI-generated patient messages |
| Rossi et al., 2024[63] | IEEE BIBM 2024 | DiabeTalk diabetes diagnostic chatbot powered by WizardLM to classify diabetes type and generate explanations | WE-LSTM classifier | Single-turn or short multi-turn interactions | Diagnostic accuracy, sensitivity, specificity and false negative rate; qualitative assessment of chatbot output |
| Saraç et al., 2025[64] | International Journal of Human–Computer Interaction | Exercise programmes generated by ChatGPT-4, ChatGPT-4o and Gemini-1.5 Pro for a standardised obese case | Expert trainer-designed exercise programme | One-time programme generation and review per model | Programme quality relative to ACSM and NASM guidelines (sets, repetitions, intensity, load progression, heart-rate zones, duration, expected BMI reduction timeline) |
| Strömel et al., 2024[65] | CHI 2024 (Conference on Human Factors in Computing Systems) | GPT-4-generated narrative text interpreting step-count data | Chart-only condition; text-only condition; combined text and chart | Interview pre-study plus single online experiment | Reflection measures; engagement; attention; open-ended feedback on narratives and visualisations |
| Szymanski et al., 2024[66] | CHI 2024 (Conference on Human Factors in Computing Systems) | Customised Food Product Nutrition Assistant with guideline-based prompts | Baseline GPT-4 with various prompt specificity levels | Single ~90-minute validation interview with 12 RDs plus prototype-refinement focus groups reported separately | Dietitian ratings of coherence, conciseness, quality and accuracy; qualitative themes on advantages, limitations, risks, design requirements and prototype feedback |
| Tayal et al., 2025 (Food)[67] | arXiv preprint | HFFood-GPT (ChatGPT-based sodium-intake assistant) and HFFood-NS (neurosymbolic system) | Within-subject comparison of HFFood-GPT and HFFood-NS | Single-session conversational interaction | Task completion; accuracy; clarity; user preference; word error rate in salt-content queries related to heart failure self-care |
| Tayal et al., 2025 (HF)[68] | arXiv preprint (SIGDIAL 2025) | ChatGPT-generated self-care dialogues about diet, exercise, and fluid intake with or without SDOH-informed reasoning | Four prompting strategies and two model versions (ChatGPT-3.5-turbo and GPT-4) | Single-round simulated dialogue generation and evaluation | Structural dialogue metrics; ratings of appropriateness; personalisation to social determinants of health; empathy; impact of explicit reasoning prompts on dialogue quality |
| Vats et al., 2025[69] | ICCTDC 2025 | AI-driven coronary artery disease risk and advisory system combining ensemble ML models, Gemini and a GPT-based chatbot | None | Single risk-prediction step and demonstration dialogues | Model performance (accuracy, precision, recall, F1-score, AUC); feasibility of integrating risk prediction and conversational guidance |
| Wali et al., 2024[70] | Discover Medicine | Heart attack risk recommender system combining a CatBoost prediction model with a BioMistral chatbot | None | Continuous interaction via web interface and wearable devices | Machine-learning performance metrics (AUC and related measures); functionality of explanations; chatbot capability for lifestyle advice and clarification of misconceptions |
| Wang et al., 2025[71] | Hypertension | Cascade Framework LLM agent (4o-Cascade) for hypertension education and clinical decision support via WeChat mini programme | Baseline ChatGPT-4o model; three practising physicians | Two-phase benchmarking and prospective external validation during a single outpatient encounter | Accuracy and comprehensiveness of educational responses; blood pressure classification accuracy; cardiovascular risk stratification; appropriateness of initial clinical decisions; patient-rated understandability, credibility and emotional support |
