# Supplementary material for: Behavior Change Content and Implementation of Large Language Model–Driven Conversational Agents in Cardiometabolic Care: Scoping Review
Source: J Med Internet Res. 2026 Jul 15;28:e89190. doi: 10.2196/89190 (PMC13372078; doi:10.2196/89190)
Supplement: Multimedia Appendix 5 [file jmir-v28-e89190-s005.docx]

**Multimedia Appendix 5: Detailed assessment of implementation-reporting transparency for large language model-driven conversational agents**

**Supplementary Table S4. Reporting of LLM implementation details across included studies (n = 38).**

Codes:

F = fully reported; P = partially reported; NR = not reported; Y = yes; N = no.

Domains:

Model description: Clearly specified model architecture, version, or API (e.g., GPT-4o, Llama 2).

Prompts and system messages: Reporting of exact prompt text (F) or general description of prompt logic or intent without full text (P).

Role and persona: Explicit definition of the agent's intended role (e.g., "Health Coach", "Educator").

Context and memory: Description of how conversation history, multi-turn state, or RAG context (retrieval mechanism) is managed.

Personalisation logic: Explanation of how user-specific data (e.g., health records, preferences, demographics) tailors the output.

Safety and oversight: Reporting of guardrails, refusal rules, human-in-the-loop mechanisms, or specific risk mitigation strategies.

Example dialogues shown: Inclusion of verbatim snippets, screenshots, or transcripts of LLM-generated text.

| **Study (first author, year)** | **Model description** | **Prompts and system messages** | **Role and persona** | **Context and memory** | **Personalisation logic** | **Safety and oversight** | **Example dialogues shown** |
| --- | --- | --- | --- | --- | --- | --- | --- |
| Abbasian et al., 2024[34] | F | P | F | F | F | P | Y |
| Aguzzi et al., 2025[35] | F | F | F | NR | NR | NR | N |
| Ahmadi et al., 2025[36] | F | NR | F | F | F | NR | N |
| Andreadis et al., 2024[37] | P | NR | F | NR | NR | NR | N |
| Antia et al., 2025[38] | P | NR | F | NR | NR | NR | N |
| Cheng et al., 2025[39] | F | F | F | F | F | F | Y |
| Chuang et al., 2025[40] | P | P | F | F | F | F | Y |
| Coleman et al., 2025[41] | F | P | F | F | P | F | Y |
| Dao et al., 2024[42] | F | F | F | F | F | P | Y |
| Đurković et al., 2025[43] | F | P | F | NR | F | F | Y |
| Elfayoumi et al., 2025[44] | F | P | F | NR | F | NR | Y |
| Gollapalli et al., 2025[45] | F | F | F | F | F | NR | Y |
| Huang et al., 2025[46] | F | P | F | NR | F | NR | Y |
| Hussain et al., 2025[47] | F | P | F | NR | NR | NR | Y |
| Jeon et al., 2025[48] | F | P | F | F | F | F | Y |
| Kelly et al., 2025[49] | F | F | F | F | NR | F | Y |
| Kozaily et al., 2023[50] | F | F | NR | F | NR | NR | Y |
| Liang et al., 2025[51] | F | P | F | F | F | F | Y |
| Meng et al., 2025 (Eval)[52] | F | NR | P | NR | NR | P | Y |
| Meng et al., 2025 (T2MD)[53] | F | P | F | F | F | NR | Y |
| Mohd Dan et al., 2025[54] | F | P | F | F | F | P | N |
| Montagna et al., 2023[55] | F | NR | F | NR | F | F | Y |
| Mustafa et al., 2025[56] | P | P | NR | NR | P | F | N |
| Neary et al., 2025[57] | P | P | F | F | F | F | Y |
| Pan, 2025[58] | F | P | F | F | F | F | Y |
| Patil et al., 2025[59] | F | P | F | NR | F | NR | Y |
| Pay et al., 2025[60] | F | F | NR | NR | NR | NR | Y |
| Ponzo et al., 2024[61] | F | F | NR | F | P | P | Y |
| Rodriguez et al., 2024[62] | F | NR | F | F | F | F | Y |
| Rossi et al., 2024[63] | F | P | F | F | F | NR | Y |
| Saraç et al., 2025[64] | F | F | F | NR | F | P | Y |
| Strömel et al., 2024[65] | F | F | F | NR | F | F | Y |
| Szymanski et al., 2024[66] | F | F | F | NR | F | F | Y |
| Tayal et al., 2025 (Food)[67] | F | F | F | F | F | F | Y |
| Tayal et al., 2025 (HF)[68] | F | F | F | NR | F | NR | Y |
| Vats et al., 2025[69] | F | P | F | NR | F | NR | Y |
| Wali et al., 2024[70] | F | P | F | F | F | P | Y |
| Wang et al., 2025[71] | F | NR | F | F | F | F | Y |

**Additional note.** For studies in which LLM use was reasonably inferable but the specific model architecture was not named, the model-description domain was coded as partially reported rather than fully reported.
